# Supplementary material for: Comparing perceived clarity of information on overdiagnosis used for breast and prostate cancer screening in England: an experimental survey
Source: BMJ Open. 2017 Aug 21;7(8):e015955. doi: 10.1136/bmjopen-2017-015955 (PMC5629687; doi:10.1136/bmjopen-2017-015955)
Supplement: Supplementary file 1 [file bmjopen-2017-015955supp001.pdf]

Appendix 1 – Sensitivity analyses: perceived clarity of overdiagnosis information: adjusted ORs, 95% CIs, p-values for categorical/ordinal variables in multivariable binary logistic regression models

	"How clear do you find this description of a risk of the test?" n (%)			
**Characteristic**	**Extremely (vs. less than extremely)**	**At least very (vs. less than very)**	**At least moderately (vs. less than moderately)**	**At least slightly (vs. not at all)**
	**Adjusted OR, 95% CI; p-value**	**Adjusted OR, 95% CI; p-value**	**Adjusted OR, 95% CI; p-value**	**Adjusted OR, 95% CI; p-value**
**Overdiagnosis information**				
Breast screening text vs. Prostate screening text	0.92, 0.65 to 1.30; .635	1.43, 1.17 to 1.75; **.001**	1.09, 0.85 to 1.40; .484	0.89, 0.64 to 1.24; .482
**Gender**				
Male vs. Female	1.03, 0.71 to 1.50; .873	0.92, 0.74 to 1.14; .447	1.20, 0.92 to 1.56; .183	1.24, 0.87 to 1.78; .243
**Ethnicity**				
White British vs. Other ethnic groups	1.26, 0.80 to 1.99; .312	1.19, 0.92 to 1.54; .197	1.35, 1.00 to 1.82; .054	1.17, 0.77 to 1.77; .464
**Marital status**				
Married or living as a couple vs. Single, widowed, divorced, or separated	0.98, 0.68 to 1.42; .909	1.16, 0.93 to 1.45; .184	1.12, 0.87 to 1.46; .382	1.03, 0.72 to 1.47; .879
**Highest level of education**	Overall: .535	Overall: .482	Overall: .075	Overall: .425

No formal qualifications	0.91, 0.50 to 1.66; .751	1.24, 0.86 to 1.79; .248	1.70, 1.07 to 2.68; .024	1.48, 0.80 to 2.75; .213
Approximately Level 1, 2, or 3	0.79, 0.52 to 1.20; .277	1.13, 0.87 to 1.46; .353	1.17, 0.86 to 1.59; .315	1.22, 0.82 to 1.84; .330
vs. Approximately Level 4				
**Social class grade**	Overall: .534	Overall: .610	Overall: .053	Overall: .260
Grade A or B	1.37, 0.79 to 2.38; .263	1.18, 0.85 to 1.64; .323	1.40, 0.95 to 2.07; .092	1.02, 0.61 to 1.70; .954
Grade C1 or C2	1.67, 0.75 to 1.83; .497	1.09, 0.85 to 1.40; .507	1.44, 1.07 to 1.94; .017	1.34, 0.89 to 2.02; .165
vs. Grade D or E				
**Personal diagnosis of cancer**				
Yes	1.23, 0.61 to 2.45; .565	1.34, 0.84 to 2.12; .218	1.20, 0.65 to 2.22; .557	1.03, 0.47 to 2.27; .934
vs. No				
**Knows someone with cancer**				
Yes	0.85, 0.59 to 1.23; .390	1.23, 0.99 to 1.53; .060	0.85, 0.65 to 1.11; .226	0.79, 0.55 to 1.13; .196
vs. No				
**Previously read a screening leaflet**				
Yes	1.30, 0.83 to 2.04; .257	1.35, 1.04 to 1.74; **.024**	1.12, 0.83 to 1.53; .461	1.11, 0.73 to 1.69; .619
vs. No				
**Previously read an NHS screening website**				
Yes	0.85, 0.54 to 1.36; .504	0.97, 0.72 to 1.30; .815	0.95, 0.65 to 1.39; .779	0.85, 0.51 to 1.42; .544
vs. No				

**Discussed screening with doctor/nurse**				
Yes	1.31, 0.87 to 1.97; .192	1.08, 0.83 to 1.39; .576	1.02, 0.74 to 1.41; .894	0.85, 0.55 to 1.30; .447
vs. No				
**Previously read or heard similar information**				
Yes	1.75, 1.19 to 2.57; **.004**	1.77, 1.40 to 2.23; **<.0005**	2.19, 1.61 to 2.98; **<.0005**	3.27, 2.08 to 5.14; **<.0005**
vs. No				

N.B. Adjusted ORs and 95% CIs are relative to a stated reference category; p-values <.05 are in bold; all predictor variables are included in each model with one exception (marked N/A in Appendix 2) due to a violation of the assumption linearity

Appendix 2 – Sensitivity analyses: perceived clarity of overdiagnosis information: adjusted ORs, 95% CIs, p-values for continuous variables in multivariable binary logistic regression models

	**"How clear do you find this description of a risk of the test?" n (%)**			
**Characteristic**	**Extremely (vs. less than extremely)**	**At least very (vs. less than very)**	**At least moderately (vs. less than moderately)**	**At least slightly (vs. not at all)**
	**Adjusted OR, 95% CI; p-value**	**Adjusted OR, 95% CI; p-value**	**Adjusted OR, 95% CI; p-value**	**Adjusted OR, 95% CI; p-value**
**Age (in years)**	1.00, 0.98 to 1.01; .642	1.00, 0.99 to 1.00; .282	1.00, 0.99 to 1.01; .329	1.00, 0.99 to 1.02; .562
**Decision-making styles**				
Avoidant score	1.01, 0.96 to 1.07; .711	1.01, 0.98 to 1.04; .713	1.03, 0.99 to 1.07; .143	0.99, 0.94 to 1.04; .611
Dependent score	0.94, 0.89 to 0.99; **.016**	0.97, 0.94 to 1.00; .052	0.98, 0.94 to 1.02; .289	1.04, 0.98 to 1.09; .176
Intuitive score	1.00, 0.94 to 1.07; .978	1.02, 0.98 to 1.06; .413	1.02, 0.98 to 1.07; .358	1.01, 0.94 to 1.07; .857
Rational score	1.15, 1.06 to 1.24; **.001**	1.06, 1.02 to 1.11; **.009**	1.03, 0.98 to 1.09; .215	1.08, 1.01 to 1.16; **.031**
Spontaneous score	1.02, 0.96 to 1.08; .574	1.03, 1.00 to 1.07; .073	N/A	1.09, 1.01 to 1.16; **.004**

N.B. Adjusted ORs and 95% CIs are per unit increase in the case of continuous variables; p-values <.05 are in bold; all predictor variables are included in each model with one exception (marked N/A) due to a violation of the assumption linearity

Appendix 3 - Numbers and percentages of participants excluded from the full sample of 2,111

**Excluded due to missing/inapplicable data on:**	**n (%)**
Reading a leaflet from the NHS about a screening test	39
Reading information about a screening test on an NHS website	15
Talking with a doctor or nurse about a screening test	18
Reading or hearing similar information about a screening test	74
Being diagnosed with cancer	20
Anyone close being diagnosed with cancer	13
Marital status	7
Education	216
Ethnicity	9
Perceived clarity of the description of a risk of the test	48
Decision-making style	36
**Total excluded**	**495 (23.4)**
**Total analysed**	**1,616**
