# Supplementary material for: Comparing perceived clarity of information on overdiagnosis used for breast and prostate cancer screening in England: an experimental survey
Source: BMJ Open. 2017 Aug 21;7(8):e015955. doi: 10.1136/bmjopen-2017-015955 (PMC5629687; doi:10.1136/bmjopen-2017-015955)
Supplement: Author's manuscript [file bmjopen-2017-015955.draft_revisions.pdf]

BMJ Open is committed to open peer review. As part of this commitment we make the peer review history of every article we publish publicly available.

When an article is published we post the peer reviewers' comments and the authors' responses online. We also post the versions of the paper that were used during peer review. These are the versions that the peer review comments apply to.

The versions of the paper that follow are the versions that were submitted during the peer review process. They are not the versions of record or the final published versions. They should not be cited or distributed as the published version of this manuscript.

BMJ Open is an open access journal and the full, final, typeset and author-corrected version of record of the manuscript is available on our site with no access controls, subscription charges or pay-per-view fees (http://bmjopen.bmj.com).

If you have any questions on BMJ Open's open peer review process please email editorial.bmjopen@bmj.com

BMJ Open

Comparing perceived clarity of information on overdiagnosis used for breast and prostate cancer screening in England

Journal:	*BMJ Open*
Manuscript ID	bmjopen-2017-015955
Article Type:	Research
Date Submitted by the Author:	12-Jan-2017
Complete List of Authors:	Ghanouni, Alex; University College London, Health Behaviour Research Centre, Department of Epidemiology & Public Health<br>Renzi, Cristina; UCL, Health Behaviour Research Centre, Department of Epidemiology and Public Health<br>McBride, Emily; University College London, Health Behaviour Research Centre<br>Waller, Jo; UCL, Health Behaviour Research Centre, Epidemiology and Public Health
**Primary Subject Heading**:	Communication
Secondary Subject Heading:	Public health
Keywords:	Decision Making, Surveys and Questionnaires, Early Detection of Cancer, Mass Screening, Medical Overuse

SCHOLARONE™ Manuscripts

FULL TITLE PAGE

Title: Comparing perceived clarity of information on overdiagnosis used for breast and prostate cancer screening in England

Manuscript type: Research article

Authors:

Alex Ghanouni[a] a.ghanouni@ucl.ac.uk

Cristina Renzi[a] c.renzi@ucl.ac.uk

Emily McBride[a] e.mcbride@ucl.ac.uk

Jo Waller[ab] j.waller@ucl.ac.uk | Tel: +44 (0)20 7679 5958 | Fax: +44 (0)20 7679 8354

Affiliations:

[a]Health Behaviour Research Centre, Department of Epidemiology and Public Health, University College London, Gower Street, London, WC1E 6BT, United Kingdom

[b]Corresponding author

Word count: Abstract: 300/300; Body: 3,040/4,000

For peer review only

ABSTRACT

Objectives: 'Overdiagnosis', detection of disease that would never have caused symptoms or death, is a public health concern due to possible psychological and physical harm. Public awareness is low and little is known about how best to explain it. This study evaluated public perceptions of widely-used information on the concept to identify scope for improving communication methods.

Design: Survey carried out by a market research company via face-to-face computer-assisted interviews.

Setting: Interviews took place in participants' homes.

Participants: 2,111 members of the general public in England aged 18-70 years began the survey; 1,616 were eligible for analysis. National representativeness was sought via demographic quota sampling.

Interventions: Participants were allocated at random to receive a brief description of overdiagnosis derived from written information used by either the NHS Breast Screening Programme or the prostate cancer screening equivalent.

Primary and secondary outcome measures: The primary outcome was how clear the information was perceived to be (extremely/very clear vs. less clear). Other measures included previous exposure to screening information, decision-making styles, and demographic characteristics (e.g. education). Binary logistic regression was used to assess predictors of perceived clarity.

Results: Overdiagnosis information from the BSP was more likely to be rated as more clear compared with the prostate screening equivalent (adjusted odds-ratio: 1.43, 95% confidence interval: 1.17 to 1.75; p=.001). Participants were more likely to perceive the information as more clear if they had previously encountered similar information (OR: 1.77, 1.40 to 2.23;

For peer review only

p<.0005) or a screening leaflet (OR: 1.35, 1.04 to 1.74; p=.024), or had a more 'rational' decision-making style (OR: 1.06, 1.02 to 1.11; p=.009).

Conclusions: Overdiagnosis information from breast screening may be a useful template for communicating the concept more generally. However, this information may be less well-suited to individuals who are less inclined to consider risks and benefits during decision-making.

Keywords: Decision Making; Surveys and Questionnaires; Early Detection of Cancer; Mass Screening; Medical Overuse

Strengths and limitations of this study:

- There has been very little research regarding how to effectively communicate the concept of overdiagnosis to lay people, which this study aims to begin to address
- It benefited from measuring a wide range of predictor variables in a large sample of the general public in which quotas were set with the aim of achieving population-representativeness
- The sample was also heterogeneous, meaning that findings may not apply equally to a specific healthcare context in which overdiagnosis may be an issue
- Information on overdiagnosis was extracted from information sources and presented separately, meaning that perceived clarity may be different when read within the original contexts
- Participants' objective understanding of the information was not measured and it is unknown how well this correlates with perceived clarity

INTRODUCTION

Overdiagnosis, defined as the detection of disease that would never have caused symptoms or death within a person's lifetime, is a topic of widespread concern in healthcare[1] since it

For peer review only

results in negative psychological consequences, overtreatment, and overutilisation of scarce resources.[2] This has led to various efforts to increase awareness of the issue and mitigate its effects.[1,3–7]

However, there is strong, consistent evidence that public awareness of overdiagnosis is markedly low, both in the UK and internationally.[8–10] For example, fewer than one in three individuals surveyed in the UK reported recognising the term and virtually no participants were able to define it accurately.[11] There is also consistent evidence that the concept is difficult to explain to lay people in a way that can be readily understood since the term is considered counterintuitive,[12,13] contradicting an established faith in the importance of early detection.[14] There is considerable scope to improve awareness and understanding but research into effective methods of communicating the concept is limited.[2]

Overdiagnosis is a prominent issue in breast and prostate cancer screening.[15] In the UK, a thorough review of written information materials was carried out for the Breast Screening Programme with the explicit aim of improving communication of the concept.[16,17] Similarly, men receiving care under the Prostate Cancer Risk Management Programme are informed about overdiagnosis, primarily by clinicians but also via associated leaflets.[18-19] These written materials offer two brief descriptions of the concept that could potentially serve as useful models for communicating it more generally.

Strategies for communicating overdiagnosis may also become apparent via a better understanding of factors associated with people's perceived understanding of the concept. We previously explored whether screening-eligible individuals' approaches to making important decisions ('decision-making styles', DMS)[20] was associated with the amount of cancer screening information leaflets they read; less 'rational' individuals who tended not to weigh up available information were less likely to have read information in leaflets used by bowel and cervical screening programmes (Ghanouni et al., under review). Since existing written information has been designed to allow individuals to consider risks and benefits, it is

For peer review only

plausible that people who are less inclined to make decisions in this way would find materials less clear. There may also be relevant associations with other DMS (e.g. 'avoidant' or 'dependent' styles) or demographic characteristics (e.g. education and previous exposure to screening information).

We carried out a large-scale survey of the general public in England to assess perceived clarity of existing, widely-used information on overdiagnosis derived from written material for breast and prostate cancer screening. We compared perceived clarity of these two pieces of information and tested for associated characteristics (specifically, DMS, demographics, and previous exposure to screening information).

METHOD

Design

Institutional ethical approval was obtained. The study design and some aspects of the measures are being described elsewhere (Ghanouni et al., under review). Data were collected between April and May, 2016, as part of Wave 3 of the Attitudes, Behaviour and Cancer UK Survey (ABACUS). A market research agency (TNS) conducted face-to-face computer-assisted interviews in people's homes, as part of their weekly omnibus survey in England. Participants were presented with information describing overdiagnosis from either the leaflet used by the NHS Breast Screening Programme or the equivalent used by the Prostate Cancer Risk Management Programme in England, allocated at random in a 1:1 ratio. Participants were told:

"We would now like to ask you some questions about leaflets on health-related topics. The NHS offers people a variety of screening tests to check for illnesses before symptoms have appeared. People offered an NHS screening test are often given a leaflet that explains the risks and benefits of having the test. The leaflet is either posted or given out by a doctor or nurse."

For peer review only

After completing items on previous exposure to screening information (described below), participants were given one of the following pieces of information:

"The test can find an illness that would never have caused a person harm. Some people will be diagnosed and treated for an illness that would never otherwise have been found and would not have become life-threatening." (From the breast screening information leaflet)[17]

"The test may make you worry by finding an illness that may never cause any symptoms or shorten your life." (From the prostate screening information sheet)[19]

Participants

Random location sampling was conducted using Census statistics and the Postcode Address File. National representativeness was achieved via quotas for demographic characteristics (e.g. employment status and gender). Participants were eligible if they were aged between 18 and 70 years.

Measures

Demographics: Principal measures were gender, ethnicity, marital status, highest level of education qualification obtained, social class grade, and age. Participants also stated whether they had been diagnosed with cancer (and what type, if applicable), or if they knew anyone who had been diagnosed.

Previous exposure to screening information: Participants were asked three questions:

1) *"Have you ever read a leaflet from the NHS about a screening test?"*

2) *"Have you ever read information about a screening test on an NHS website?"*

3) *"Have you ever talked with a doctor or nurse about a screening test?"*

(Response options: *"Yes"*, *"no"*, *"not sure"*).

For peer review only

If eligible for either cervical, breast, or bowel screening programmes in England and anticipated not to find the question distressing, participants were asked to state their previous screening history using up to four items based on the Precaution Adoption Process Model (PAPM)[21] e.g.:

"The next set of questions are about breast screening. The NHS breast screening programme invites women to have regular mammograms (x-rays of their breasts). "Which one of the following best describes you?"

Response options were: *"I have never heard of breast screening, I have heard of breast screening but have never been invited, I have been invited to breast screening but have never been, I have been invited to breast screening but have not been every time I was invited, I have been invited to breast screening and have been every time I was invited"*

Since prostate screening in England is carried out via direct contact with clinicians rather than an organised programme, previous participation was assessed in the following way among eligible participants:

"Men can have a blood test, called a prostate-specific antigen (PSA) test, to look for a protein in their blood that can be an indicator of prostate cancer. Which of the following best describes you?"

Response options were: *"I have never heard of a PSA test, I have heard of a PSA test but have never discussed it with a doctor or nurse, I have discussed a PSA test with a doctor or nurse but have never had it, I have discussed a PSA test with a doctor or nurse and have had the test, I have had the PSA test but I have never discussed it with a doctor or nurse"*

Participants were not asked these questions if they had previously been diagnosed with the applicable type of cancer, or if they were ineligible in terms of their gender or age (i.e. not aged 18-64 years for cervical screening questions, 47-70 years for breast screening

For peer review only

questions, 58-70 for bowel screening questions, or 45-70 years for prostate screening questions).

Decision-making styles: Participants were informed:

"*We would like to start by asking you some questions about how you make important decisions. I am going to read out some statements describing how individuals go about making important decisions and I would like you to tell me how much you agree or disagree with each one."*

Tendencies towards using each decision-making style were measured using 25 previously developed items that have been reported to have content, concurrent, and construct validity.[20] These relate to five subscales, measured with five items each: 'Avoidant' (e.g. *"I postpone decision making whenever possible"*), 'dependent' (e.g. *"I rarely make important decisions without consulting other people"*), 'intuitive' (e.g. *"I generally make decisions that feel right to me"*), 'rational' (e.g. *"My decision-making requires careful thought"*), and 'spontaneous' (e.g. *"I make quick decisions"*). Item order was randomised for each participant. Reliability was good for all five subscales within the analysed sample (Cronbach's α: 0.71 to 0.81).

Overdiagnosis information: Text on overdiagnosis was followed by the question, *"How clear do you find this description of a risk of the test?"*. Response options were: *"Extremely clear"*, *"very clear"*, *"moderately clear"*, *"slightly clear"*, and *"not at all clear"*. Participants were also asked *"Have you ever read or heard similar information about a screening test?"* (response options: *"Yes"*, *"no"*, *"not sure"*).

Piloting

Piloting aimed to ensure that the survey was acceptable and manageable for participants, and that items were comprehensible. This consisted of a series of cognitive interviews[22] with 11 members of the general public and a 'soft-launch' of the survey to 431 participants.

For peer review only

Analysis

Participants were excluded if data were missing, if they responded *“not sure”* or *“don’t know”*, to any item, or had a non-ordinal level of education.

Items relating to each decision-making style were scored from one to five and summed to create an overall score (higher scores represented a greater tendency to use a given approach to decision-making) and highest level of education was categorised based on Levels 1-4 used by the Office of National Statistics.[23] Social class grade was categorised as *“Grades A or B”*, *“Grades C1 or C2”*, or *“Grades D or E”*. Ethnicity and marital status were dichotomised into *“White British”* vs. *“Other ethnic groups”* and *“Married or living as a couple”* vs. *“Single, widowed, divorced, or separated”*, respectively.

Descriptive statistics were used to summarise the analysed sample overall and within each response category of perceived clarity of the overdiagnosis information. Responses on PAPM items were also summarised (previous screening participation vs. no previous participation) separately for the four screening modalities for all individuals who completed them (i.e. excluding those who were not eligible to be asked the question and those who declined to answer).

The main analysis tested the null hypothesis that overdiagnosis text (from breast vs. prostate information), demographics characteristics, previous exposure to screening information, and scores on the five DMS scales were not associated with the primary outcome (perceived clarity of information). The assumption of proportional odds was violated (test of parallel lines: p=.005) and so binary logistic regression was used, in which the outcome was dichotomised in a way that the authors considered most conceptually meaningful (*“extremely”* or *“very clear”* vs. *“moderately”*, *“slightly”* or *“not at all clear”*), instead of an ordinal logistic model.

Demographic predictor variables used were gender, marital status, ethnicity, highest level of education, social class grade, previous diagnosis of cancer, and knowing someone

For peer review only

diagnosed with cancer. Variables related to previous exposure to screening information consisted of having previously i) read a leaflet about screening, ii) read an NHS website about screening, iii) discussed screening with a doctor or nurse, and iv) read or heard similar information. Scores on the five DMS subscales were also included. The remaining predictor variable was text on overdiagnosis. There was minimal (multi)collinearity between these variables; Variance Inflation Factors were ≤1.830 and there were no violations of the assumption of linearity (Box-Tidwell procedure: all p-values >.114).

Adjusted odds-ratios (ORs) for rating the information as ‘extremely’ or ‘very clear’ vs. reporting a lower category of perceived clarity are reported with 95% confidence intervals and p-values, alongside descriptive statistics.

RESULTS

Participant characteristics

Interviews were carried out with 2,111 participants; 495 were excluded due to missing or inapplicable data, leaving a total of 1,616 in the main analysis. Percentages of participants who reported previously participating in screening were 75.2%, 80.5%, 60.9%, and 16.6% for cervical, breast, bowel, and prostate screening, respectively (n=732; n=343; n=407; n=349). Other sample characteristics are described in Table 1 and Table 2.

Predictors of rating the information on overdiagnosis as extremely/very clear

Participants were more likely to rate the breast overdiagnosis information as extremely or very clear (vs. moderately, slightly, or not at all clear) compared with the relevant information for prostate screening. Previous exposure to screening information was also associated: participants who had read a screening leaflet or had read or heard similar information before were more likely to rate the information as extremely/very clear. However, previously reading an NHS website about screening and talking to a health care professional about screening was not associated.

For peer review only

Rational decision-making style scores were associated: participants with higher scores were more likely to rate the information as extremely/very clear. There was moderate evidence against the null hypothesis for a relationship in the opposite direction in the case of the dependent decision-making style. Trends are illustrated in Figure 1. There were no other associations between other variables and the outcome (Table 1 and Table 2).

DISCUSSION

In this survey of the general public in England, less than half of the sample rated either description of overdiagnosis as either extremely or very clear (38.4% and 46.8% for prostate and breast screening respectively), consistent with previous research reporting that the concept is challenging for lay people to understand.[12,13] To date, there has been little experimental research exploring the most effective methods of communicating overdiagnosis and related concepts;[2] previous research has assessed effects of manipulating disease terminology on treatment preferences (e.g. 'abnormal cells' as an alternative to 'ductal carcinoma in situ' in the case of breast cancer)[24] and effects of information formats on comprehension of overdiagnosis information in screening (text, fact boxes, and visual aids).[25] To our knowledge, this is the first study to compare how clear the public perceived two existing pieces of widely used information on overdiagnosis to be.

The information from the breast screening leaflet was somewhat more likely to be rated as clear than the equivalent information for prostate screening. This may be attributable to the intensive multistage development process that underpinned the revised breast information leaflet, consisting of a 'citizens' jury', expert input, cognitive testing with prospective service users, and combining feedback from experts and lay people. There are two main practical implications of this finding. Firstly, the information perceived as less clear in the prostate cancer screening leaflet could be adapted it to be more consistent with the breast screening information. In principle, written information is less important for prostate screening, since it is discussed primarily face-to-face with a healthcare professional, whereas the Breast

For peer review only

Screening Programme is run on the basis of posting out written information leaflets, without involvement from a clinician. However, we found that talking with a doctor or nurse was not associated with perceived clarity, suggesting an important role for written material and potentially scope for improving face-to-face communication as well. Second, it may be useful to adapt overdiagnosis information from breast screening when attempting to describe the concept in other contexts. For example, the NHS Abdominal Aortic Aneurysm Programme also addresses overdiagnosis ("…around 54 out of every 10,000 men screened will eventually have surgery to repair an aneurysm. On average, one of these 54 men will not survive the operation but their aneurysm may never have burst if left untreated.");[26] it may be informative to assess how well this information is understood compared with text from breast screening.

There were also associations between greater perceived clarity of the information and previous exposure to screening leaflets. Although unsurprising, this is reassuring in that it provides some evidence that the leaflets are providing enough background to facilitate understanding of information on overdiagnosis at a later stage (48.6% of participants rated the information extremely/very clear among those who had read a leaflet vs 35.1% of those who had not). This was evident after controlling for exposure to similar information, (which was also associated) suggesting that it was not due to merely reencountering previously seen information.

Similar to our previous study on engagement with written screening information (Ghanouni et al., under review), there was an association with a 'rational' decision-making style. Participants who were more inclined to make decisions in this way were more likely to find the information extremely or very clear, although it was surprising that this characteristic was associated with perceived clarity, whereas education was not. This finding suggests that the information is not serving individuals as well if they tend not to make decisions in this way. It may be beneficial to explore ways of describing the concept that require less deliberation, such as a more heavily summarised 'gist'[27] or an anecdotal 'narrative' approach,[28,29]

For peer review only

describing a personal experience. There was also (weaker) evidence for a negative association between dependent decision-making and perceived clarity. This may be mitigatable by providing clear instruction on who an individual can contact for further information (e.g. a primary care provider).

This study has limitations. First, although we added some background information regarding the aims and design of screening programmes, information on overdiagnosis was removed from its original context, which includes more detail on practicalities and outcomes of screening. Second, we asked participants to rate perceived clarity of information, rather than questions testing objective understanding. These two factors mean that if participants had encountered the information outside of a study context, they may have had better comprehension (or possibly worse comprehension, in the presence of competing information points) and had more or less realistic impressions of how clear they found it. Future research could quantify the extent to which the descriptions used are understood correctly by more objective standards. Finally, the study aimed to determine predictors of perceived clarity in the general public, meaning that the sample was heterogeneous in terms of eligibility for specific screening modalities and the extent to which they were at risk of overdiagnosis in other healthcare contexts. In areas with a focus on communicating with a more specific group, it may be beneficial to test whether these findings replicate and whether other factors are relevant.

In conclusion, we found that information on overdiagnosis from the Breast Screening Programme in England is more likely to be rated as clearer than equivalent information from the Prostate Cancer Risk Management Programme and may be a valuable template for efforts to communicate the concept (including possibly revising PCRMP materials). Also, people who were less ‘rational’ and more ‘dependent’ in their decision-making may find existing information more challenging, suggesting that other descriptions may be appropriate for these individuals. Future research aiming to improve methods of communicating overdiagnosis information could assess possible descriptions of overdiagnosis in terms of

For peer review only

objective knowledge and test for associations within specific groups who may find the concept relevant.

Funding: The current study was supported by a programme grant from Cancer Research UK awarded to Prof Jane Wardle (C1418/A14134). Dr Jo Waller is supported by a Career Development Fellowship from Cancer Research UK (C7492/A17219). Cancer Research UK was not involved in the design of this study; the collection, analysis, or interpretation of the results; in the writing of the manuscript; or in the decision to submit for publication.

Competing interests: None declared.

Contributors: AG, CR, and JW conceived and designed the study. AG and EM analysed the data. AG, CR, and JW participated in the interpretation. All authors drafted the manuscript, participated in critical revision, and approved the final version.

Data sharing: No additional data are available.

REFERENCES

1 Glasziou P, Moynihan R, Richards T, Godlee F. Too much medicine; too little care. BMJ 2013;347:f4247.

2 McCaffery KJ, Jansen J, Scherer LD, et al. Walking the tightrope: communicating overdiagnosis in modern healthcare. BMJ 2016;352:i348.

3 Malhotra A, Maughan D, Ansell J, et al. Choosing Wisely in the UK: the Academy of Medical Royal Colleges' initiative to reduce the harms of too much medicine. BMJ 2015;350:h2308.

4 Journal of the American Medical Association. Less is More: http://jamanetwork.com/collections/6017/less-is-more Accessed: 28th November, 2016.

5 Calderwood C. Chief Medical Officer's annual report 2014-15. 2016.

For peer review only

http://www.gov.scot/Publications/2016/01/3745 Accessed: 28th November, 2016.

6 Hersch J, Barratt A, Jansen J, et al. Use of a decision aid including information on overdetection to support informed choice about breast cancer screening: a randomised controlled trial. Lancet 2015;385:1642-52.

7 Buchbinder R, Gross DP, Werner EL, Hayden JA. Understanding the characteristics of effective mass media campaigns for back pain and methodological challenges in evaluating their effects. Spine (Phila Pa 1976) 2008;33:74–80.

8 Moynihan R, Nickel B, Hersch J, et al. Public opinions about overdiagnosis: A national community survey. PLoS One 2015;10:e0125165.

9 Wegwarth O, Gigerenzer G. Less is more: Overdiagnosis and overtreatment: Evaluation of what physicians tell their patients about screening harms. JAMA Intern Med 2013;173:2086-7.

10 Schwartz LM, Woloshin S, Sox HC, Fischhoff B, Welch HG. US women's attitudes to false positive mammography results and detection of ductal carcinoma in situ: cross sectional survey. BMJ 2000;320:1635–40.

11 Ghanouni A, Meisel SF, Renzi C, Wardle J, Waller J. Public definitions of the term 'overdiagnosis' in the UK. BMJ Open 2016;6:e010723.

12 Hersch J, Jansen J, Barratt A, et al. Women's views on overdiagnosis in breast cancer screening: a qualitative study. BMJ 2013;346:f158.

13 Waller J, Douglas E, Whitaker KL, Wardle J. Women's responses to information about overdiagnosis in the UK breast cancer screening programme: a qualitative study. BMJ Open 2013;3:e002703.

14 Welch H, Schwartz L, Woloshin S. Overdiagnosed: making people sick in the pursuit of health. Beacon Press, 2011.

For peer review only

15 House of Commons Science and Technology Committee. National Health Screening. Third Report of Session 2014-15. 2015. http://www.parliament.uk/business/committees/committees-a-z/commons-select/science-and-technology-committee/inquiries/parliament-2010/national-health-screening Accessed: 28th November, 2016.

16 Forbes LJ, Ramirez AJ, Expert group on Information about Breast Screening. Offering informed choice about breast screening. J Med Screen 2014;21:194–200.

17 National Breast Screening Programme. NHS Breast Screening: Helping you decide. 2013. https://www.gov.uk/government/publications/breast-screening-helping-women-decide Accessed: 28th November, 2016.

18 Public Health England. Prostate cancer risk management programme (PCRMP): benefits and risks of PSA testing. 2016. https://www.gov.uk/government/publications/prostate-cancer-risk-management-programme-psa-test-benefits-and-risks/prostate-cancer-risk-management-programme-pcrmp-benefits-and-risks-of-psa-testing Accessed: 28th November, 2016.

19 Public Health England. PSA testing and prostate cancer: advice for well men aged 50 and over. 2016. https://www.gov.uk/government/publications/prostate-specific-antigen-testing-description-in-brief Accessed: 28th November, 2016.

20 Scott SG, Bruce RA. Decision-making style: The development and assessment of a new measure. Educ Psychol Meas 1995;55:818–31.

21 Weinstein ND. The precaution adoption process. Health Psychol 1988;7:355–86.

22 Willis GB, Artino AR. What do our respondents think we're asking? Using cognitive interviewing to improve medical education surveys. J Grad Med Educ 2013;5:353–6.

23 Office of National Statistics. 2011 Census: Key Statistics for England and Wales,

For peer review only

March 2011. 2012. http://www.ons.gov.uk/ons/rel/census/2011-census/key-statistics-for-local-authorities-in-england-and-wales/stb-2011-census-key-statistics-for-england-and-wales.html Accessed: 28th November, 2016.

24 Nickel B, Barratt A, Hersch J, Moynihan R, Irwig L, McCaffery K. How different terminology for ductal carcinoma in situ (DCIS) impacts women's concern and management preferences: A qualitative study. Breast 2015;24:673–9.

25 Petrova D, Garcia-Retamero R, Cokely ET. Understanding the harms and benefits of cancer screening: a model of factors that shape informed decision making. Med Decis Making 2015;35:847-58.

26 Public Health England. Abdominal aortic aneurysm (AAA) screening, a free test for men aged 65 and over. 2015. https://www.gov.uk/government/publications/abdominal-aortic-aneurysm-screening-invitation-leaflet Accessed: 28th November, 2016.

27 Smith SG, Raine R, Obichere A, Wolf MS, Wardle J, von Wagner C. The effect of a supplementary ('gist-based') information leaflet on colorectal cancer knowledge and screening intention: a randomized controlled trial. J Behav Med 2015;38:261–72.

28 Green MC. Narratives and cancer communication. J Commun 2006;56:S163–83.

29 Hinyard LJ, Kreuter MW. Using narrative communication as a tool for health behavior change: a conceptual, theoretical, and empirical overview. Health Educ Behav 2007;34:777–92.

For peer review only

Table 1 – Perceived clarity of overdiagnosis information: descriptive statistics for categorical/ordinal variables, adjusted ORs, 95% CIs, p-values for variables in the multivariable binary logistic regression model

		"How clear do you find this description of a risk of the test?" n (%)					Adjusted OR, 95% CI; p-value
Characteristic	Total (N=1,616)	Extremely clear (n=148; 9.2%)	Very clear (n=542; 33.5%)	Moderately clear (n=592; 36.6%)	Slightly clear (n=173; 10.7%)	Not at all clear (n=161; 10.0%)	Extremely/Very clear (vs. Not/Slightly/Moderately)
**Overdiagnosis information**							
Breast screening text	850	75 (8.8)	321 (37.8)	285 (33.5)	81 (9.5)	88 (10.4)	1.43, 1.17 to 1.75; **.001**
vs. Prostate screening text	766	73 (9.5)	221 (28.9)	307 (40.1)	92 (12.0)	73 (9.5)	
**Gender**							
Male	748	66 (8.8)	228 (30.5)	303 (40.5)	81 (10.8)	70 (9.4)	0.92, 0.74 to 1.14; .447
vs. Female	868	82 (9.4)	314 (36.2)	289 (33.3)	92 (10.6)	91 (10.5)	
**Ethnicity**							
White British	1,229	116 (9.4)	425 (34.6)	447 (36.4)	121 (9.8)	120 (9.8)	1.19, 0.92 to 1.54; .197
vs. Other ethnic groups	387	32 (8.3)	117 (30.2)	145 (37.5)	52 (13.4)	41 (10.6)	
**Marital status**							
Married or living as a couple	987	92 (9.3)	344 (34.9)	355 (36)	101 (10.2)	95 (9.6)	1.16, 0.93 to 1.45; .184
vs. Single, widowed, divorced, or separated	629	56 (8.9)	198 (31.5)	237 (37.7)	72 (11.4)	66 (10.5)	

For peer review only

**Highest level of education**							Overall p-value: .482
No formal qualifications	275	24 (8.7)	97 (35.3)	107 (38.9)	25 (9.1)	22 (8.0)	1.24, 0.86 to 1.79; .248
Approximately Level 1, 2, or 3	858	68 (7.9)	297 (34.6)	312 (36.4)	97 (11.3)	84 (9.8)	1.13, 0.87 to 1.46; .353
vs. Approximately Level 4	483						
**Social class grade**							Overall p-value: .610
Grade A or B	351	41 (11.7)	117 (33.3)	122 (34.8)	31 (8.8)	40 (11.4)	1.18, 0.85 to 1.64; .323
Grade C1 or C2	786	71 (9.0)	267 (34.0)	298 (37.9)	80 (10.2)	70 (8.9)	1.09, 0.85 to 1.40; .507
vs. Grade D or E	479	36 (7.5)	158 (33.0)	172 (35.9)	62 (12.9)	51 (10.6)	
**Personal diagnosis of cancer**							
Yes	86	11 (12.8)	36 (41.9)	25 (29.1)	6 (7.0)	8 (9.3)	1.34, 0.84 to 2.12; .218
vs. No	1,530	137 (9.0)	506 (33.1)	567 (37.1)	167 (10.9)	153 (10.0)	
**Knows someone with cancer**							
Yes	946	89 (9.4)	348 (36.8)	313 (33.1)	97 (10.3)	99 (10.5)	1.23, 0.99 to 1.53; .060
vs. No	670	59 (8.8)	194 (29.0)	279 (41.6)	76 (11.3)	62 (9.3)	
**Previously read a screening leaflet**							
Yes	907	101 (11.1)	340 (37.5)	298 (32.9)	87 (9.6)	81 (8.9)	1.35, 1.04 to 1.74; **.024**
vs. No	709	47 (6.6)	202 (28.5)	294 (41.5)	86 (12.1)	80 (11.3)	

For peer review only

**Previously read an NHS screening website**							
Yes	275	33 (12.0)	105 (38.2)	88 (32.0)	23 (8.4)	26 (9.5)	0.97, 0.72 to 1.30; .815
vs. No	1,341	115 (8.6)	437 (32.6)	504 (37.6)	150 (11.2)	135 (10.1)	
**Discussed screening with doctor/nurse**							
Yes	535	67 (12.5)	200 (37.4)	172 (32.1)	46 (8.6)	50 (9.3)	1.08, 0.83 to 1.39; .576
vs. No	1,081	81 (7.5)	342 (31.6)	420 (38.9)	127 (11.7)	111 (10.3)	
**Previously read or heard similar information**							
Yes	584	78 (13.4)	239 (40.9)	189 (32.4)	49 (8.4)	29 (5.0)	1.77, 1.40 to 2.23; **<.0005**
vs. No	1,032	70 (6.8)	303 (29.4)	403 (39.1)	124 (12.0)	132 (12.8)	

N.B. Adjusted ORs and 95% CIs are relative to a stated reference category; p-values <.05 are in bold; all predictor variables are included in the model

For peer review only

Table 2 – Perceived clarity of overdiagnosis information: descriptive statistics for continuous variables, adjusted ORs, 95% CIs, p-values for variables in the multivariable binary logistic regression model

		"How clear do you find this description of a risk of the test?" M (SD)					Adjusted OR, 95% CI; p-value
**Characteristic**	**Total (n=1,616)**	**Extremely clear (n=148; 9.2%)**	**Very clear (n=542; 33.5%)**	**Moderately clear (n=592; 36.6%)**	**Slightly clear (n=173; 10.7%)**	**Not at all clear (n=161; 10.0%)**	**Extremely/Very clear (vs. Not/Slightly/Moderately)**
**Age (in years)**	43.7 (15.7)	45.4 (14.8)	44.3 (16)	43.3 (15.9)	43.2 (15.7)	42.4 (14.7)	1.00, 0.99 to 1.00; .282
**Decision-making styles**							
Avoidant score	13.8 (3.9)	13.3 (4.4)	13.8 (3.8)	13.9 (3.9)	13.7 (3.9)	13.5 (3.8)	1.01, 0.98 to 1.04; .713
Dependent score	16.9 (3.5)	16.2 (4)	16.8 (3.5)	17.0 (3.4)	17.4 (3.5)	16.5 (3.4)	0.97, 0.94 to 1.00; .052
Intuitive score	18.7 (2.8)	18.8 (3.4)	18.9 (2.7)	18.6 (2.7)	18.7 (2.7)	18.3 (2.6)	1.02, 0.98 to 1.06; .413
Rational score	19.7 (2.6)	20.4 (2.7)	19.7 (2.6)	19.6 (2.6)	19.7 (2.8)	19.4 (2.6)	1.06, 1.02 to 1.11; **.009**
Spontaneous score	14.7 (3.7)	14.5 (4.1)	14.9 (3.6)	14.9 (3.6)	14.5 (3.8)	14.0 (3.7)	1.03, 1.00 to 1.07; .073

N.B. Adjusted ORs and 95% CIs are per unit increase; p-values <.05 are in bold; all predictor variables are included in the model

For peer review only

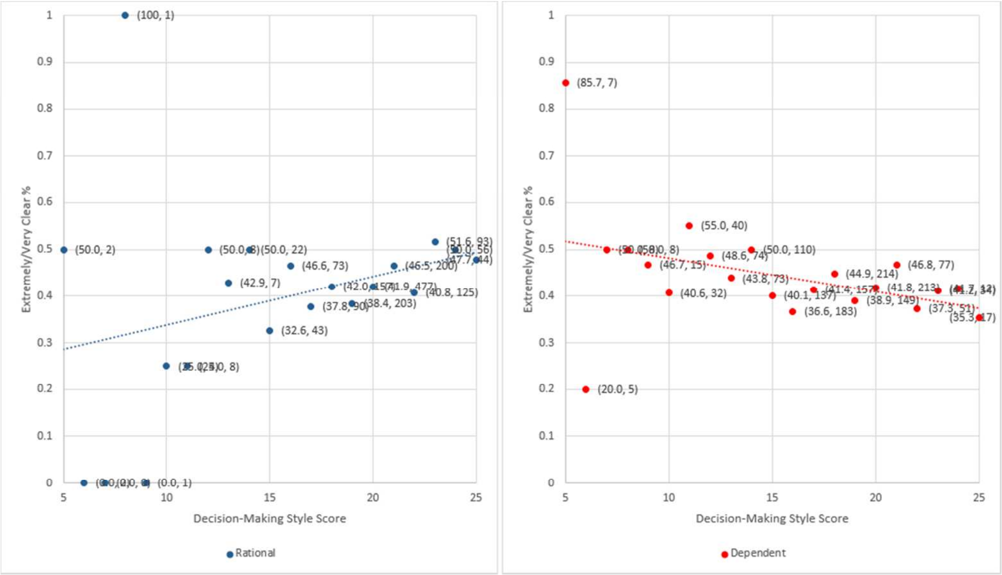

Figure 1 – Proportion of participants who perceived the information as extremely/very clear within each possible score level for rational and dependent decision-making styles (percentage and total number of participants in brackets)

263x151mm (96 x 96 DPI)

eview only

BMJ Open

Comparing perceived clarity of information on overdiagnosis used for breast and prostate cancer screening in England: An experimental survey

Journal:	*BMJ Open*
Manuscript ID	bmjopen-2017-015955.R1
Article Type:	Research
Date Submitted by the Author:	31-May-2017
Complete List of Authors:	Ghanouni, Alex; University College London, Research Department of Behavioural Science and Health<br>Renzi, Cristina; UCL, Research Department of Behavioural Science and Health<br>McBride, Emily; University College London, Research Department of Behavioural Science and Health<br>Waller, Jo; UCL, Research Department of Behavioural Science and Health
**Primary Subject Heading**:	Communication
Secondary Subject Heading:	Public health
Keywords:	Decision Making, Surveys and Questionnaires, Early Detection of Cancer, Mass Screening, Medical Overuse

SCHOLARONE™ Manuscripts

For peer review only

FULL TITLE PAGE

Title: Comparing perceived clarity of information on overdiagnosis used for breast and prostate cancer screening in England: An experimental survey

Manuscript type: Research article

Authors:

Alex Ghanouni[a] a.ghanouni@ucl.ac.uk

Cristina Renzi[a] c.renzi@ucl.ac.uk

Emily McBride[a] e.mcbride@ucl.ac.uk

Jo Waller[ab] j.waller@ucl.ac.uk | Tel: +44 (0)20 7679 5958 | Fax: +44 (0)20 7679 8354

Affiliations:

[a]Research Department of Behavioural Science and Health, University College London, Gower Street, London, WC1E 6BT, United Kingdom

[b]Corresponding author

Word count: Abstract: 296/300; Body: 3,456/4,000

For peer review only - http://bmjopen.bmj.com/site/about/guidelines.xhtml

ABSTRACT

Objectives: 'Overdiagnosis', detection of disease that would never have caused symptoms or death, is a public health concern due to possible psychological and physical harm but little is known about how best to explain it. This study evaluated public perceptions of widely-used information on the concept to identify scope for improving communication methods.

Design: Experimental survey carried out by a market research company via face-to-face computer-assisted interviews.

Setting: Interviews took place in participants' homes.

Participants: 2,111 members of the general public in England aged 18-70 years began the survey; 1,616 were eligible for analysis. National representativeness was sought via demographic quota sampling.

Interventions: Participants were allocated at random to receive a brief description of overdiagnosis derived from written information used by either the NHS Breast Screening Programme or the prostate cancer screening equivalent.

Primary and secondary outcome measures: The primary outcome was how clear the information was perceived to be (extremely/very clear vs. less clear). Other measures included previous exposure to screening information, decision-making styles, and demographic characteristics (e.g. education). Binary logistic regression was used to assess predictors of perceived clarity.

Results: Overdiagnosis information from the BSP was more likely to be rated as more clear compared with the prostate screening equivalent (adjusted odds-ratio: 1.43, 95% confidence interval: 1.17 to 1.75; p=.001). Participants were more likely to perceive the information as more clear if they had previously encountered similar information (OR: 1.77, 1.40 to 2.23; p<.0005) or a screening leaflet (OR: 1.35, 1.04 to 1.74; p=.024), or had a more 'rational' decision-making style (OR: 1.06, 1.02 to 1.11; p=.009).

For peer review only - http://bmjopen.bmj.com/site/about/guidelines.xhtml

Conclusions: Overdiagnosis information from breast screening may be a useful template for communicating the concept more generally. However, this information may be less well-suited to individuals who are less inclined to consider risks and benefits during decision-making.

Keywords: Decision Making; Surveys and Questionnaires; Early Detection of Cancer; Mass Screening; Medical Overuse

Strengths and limitations of this study:

- There has been very little research regarding how to effectively communicate the concept of overdiagnosis to lay people, which this study aims to begin to address
- It benefited from measuring a wide range of predictor variables in a large sample of the general public in which quotas were set with the aim of achieving population-representativeness
- The sample was also heterogeneous, meaning that findings may not apply equally to a specific healthcare context in which overdiagnosis may be an issue
- Information on overdiagnosis was extracted from information sources and presented separately, meaning that perceived clarity may be different when read within the original contexts
- Participants' objective understanding of the information was not measured and it is unknown how well this correlates with perceived clarity

INTRODUCTION

Overdiagnosis, defined as the detection of disease that would never have caused symptoms or death within a person's lifetime, is a topic of widespread concern in healthcare[1] since it results in negative psychological consequences, overtreatment, and overutilisation of scarce resources.[2] This has led to various efforts to communicate the issue to patients and the

For peer review only - http://bmjopen.bmj.com/site/about/guidelines.xhtml

public, increasing awareness so that it can be factored into decision-making around health.[1,3–7]

However, these efforts have proved challenging. For example, there is strong, consistent evidence that public awareness of overdiagnosis remains markedly low, both in the UK and internationally.[8–10] Fewer than one in three individuals surveyed in the UK reported recognising the term and virtually no participants were able to define it accurately.[11] This demonstrates that there is considerable scope for improving understanding of the concept, although research into effective communication methods is limited.[2] The available studies have also shown that the concept is difficult to explain in a way that lay people find clear. The term is often considered counterintuitive[12,13] since it contradicts an established faith in the importance of early detection.[14] A perceived lack of clarity of information on overdiagnosis is problematic since it is likely to result in feelings of uncertainty, contributing to decisional conflict.[15]

Breast and prostate cancer screening are two notable contexts in which overdiagnosis is a prominent issue.[16] In the UK, a thorough review of written information materials was carried out for the Breast Screening Programme with the explicit aim of improving communication of the concept.[17,18] Similarly, men receiving care under the Prostate Cancer Risk Management Programme are informed about overdiagnosis, primarily by clinicians but also via associated leaflets.[19,20] These written materials offer two brief descriptions of the concept that could potentially serve as models for communicating it more generally (e.g. to screening invitees or the wider public).

Strategies for communicating overdiagnosis may also become apparent via a better understanding of factors associated with perceived clarity of explanations, such as people's approaches to making important decisions ('decision-making styles'; DMS).[21] We previously explored whether this was associated with the amount of cancer screening information leaflets that screening-eligible individuals had read; less 'rational' participants who tended

For peer review only - http://bmjopen.bmj.com/site/about/guidelines.xhtml

not to weigh up available information were less likely to have read information in leaflets used by bowel and cervical screening programmes.[22] Since existing written information has been designed to allow individuals to consider risks and benefits, it is plausible that people who are less inclined to make decisions in this way would find materials less clear. There may also be relevant associations with other DMS (e.g. 'avoidant' or 'dependent' styles) or demographic characteristics (e.g. education and previous exposure to screening information).

We carried out a large-scale experimental survey of the general public in England to assess perceived clarity of existing, widely-used information on overdiagnosis derived from written material for breast and prostate cancer screening. We compared perceived clarity of these two pieces of information and tested for associated characteristics (specifically, DMS, demographics, and previous exposure to screening information).

METHOD

Design

Institutional ethical approval was obtained from the University College London Research Ethics Committee (5771/002). The study design and some aspects of the measures have also been described elsewhere.[22] Data were collected between April and May, 2016, as part of Wave 3 of the Attitudes, Behaviour and Cancer UK Survey (ABACUS). A market research agency (TNS) conducted face-to-face computer-assisted interviews in people's homes, as part of their weekly omnibus survey in England. Participants were presented with information describing overdiagnosis from either the leaflet used by the NHS Breast Screening Programme or the equivalent used by the Prostate Cancer Risk Management Programme in England, allocated at random in a 1:1 ratio. Participants were told:

"We would now like to ask you some questions about leaflets on health-related topics. The NHS offers people a variety of screening tests to check for illnesses before symptoms have appeared. People offered an NHS screening test are often given a leaflet that explains the

For peer review only - http://bmjopen.bmj.com/site/about/guidelines.xhtml

risks and benefits of having the test. The leaflet is either posted or given out by a doctor or nurse."

After completing items on previous exposure to screening information (described below), participants were given one of the following pieces of information:

"The test can find an illness that would never have caused a person harm. Some people will be diagnosed and treated for an illness that would never otherwise have been found and would not have become life-threatening." (From the breast screening information leaflet)[18]

"The test may make you worry by finding an illness that may never cause any symptoms or shorten your life." (From the prostate screening information sheet)[20]

The provided descriptions consisted of all the text relating to overdiagnosis in the prostate screening leaflet (as it existed at the time the survey was designed) and all relevant conceptual information from the breast screening leaflet (i.e. omitting overlapping but context-specific information on ductal carcinoma in situ and ratios of breast cancer deaths prevented to overdiagnosed cases). Text that reiterated the information above with slightly different wording was also omitted. The breast screening leaflet is freely available online; the original prostate screening leaflet is available on request.

Participants

Random location sampling was conducted using Census statistics and the Postcode Address File. National representativeness was achieved via quotas for demographic characteristics (e.g. employment status and gender). Participants were eligible if they were aged between 18 and 70 years. The total number of participants approached for this study was determined by funding constraints for the broader ABACUS survey.

Measures

For peer review only - http://bmjopen.bmj.com/site/about/guidelines.xhtml

For peer review only

Demographics: Principal measures were gender, ethnicity, marital status, highest level of education qualification obtained, and age. We also measured social class grade (a widely used classification of socioeconomic status based on type of employment).[23] Participants also stated whether they had been diagnosed with cancer (and what type, if applicable), or if they knew anyone who had been diagnosed.

Previous exposure to screening information: Participants were asked three questions:

1) *“Have you ever read a leaflet from the NHS about a screening test?”*

2) *“Have you ever read information about a screening test on an NHS website?”*

3) *“Have you ever talked with a doctor or nurse about a screening test?”*

(Response options: *“Yes”*, *“no”*, *“not sure”*).

If eligible for either cervical, breast, or bowel screening programmes in England and anticipated not to find the question distressing, participants were asked to state their previous screening history using up to four items based on the Precaution Adoption Process Model (PAPM)[24] e.g.:

“The next set of questions are about breast screening. The NHS breast screening programme invites women to have regular mammograms (x-rays of their breasts). “Which one of the following best describes you?”

Response options were: *“I have never heard of breast screening, I have heard of breast screening but have never been invited, I have been invited to breast screening but have never been, I have been invited to breast screening but have not been every time I was invited, I have been invited to breast screening and have been every time I was invited”*

Since prostate screening in England is carried out via direct contact with clinicians rather than an organised programme, previous participation was assessed in the following way among eligible participants:

For peer review only - http://bmjopen.bmj.com/site/about/guidelines.xhtml

"Men can have a blood test, called a prostate-specific antigen (PSA) test, to look for a protein in their blood that can be an indicator of prostate cancer. Which of the following best describes you?"

Response options were: *"I have never heard of a PSA test, I have heard of a PSA test but have never discussed it with a doctor or nurse, I have discussed a PSA test with a doctor or nurse but have never had it, I have discussed a PSA test with a doctor or nurse and have had the test, I have had the PSA test but I have never discussed it with a doctor or nurse"*

Participants were not asked these questions if they had previously been diagnosed with the applicable type of cancer, or if they were ineligible in terms of their gender or age (i.e. not aged 18-64 years for cervical screening questions, 47-70 years for breast screening questions, 58-70 for bowel screening questions, or 45-70 years for prostate screening questions).

Decision-making styles: Participants were informed:

"*We would like to start by asking you some questions about how you make important decisions. I am going to read out some statements describing how individuals go about making important decisions and I would like you to tell me how much you agree or disagree with each one."*

Tendencies towards using each decision-making style were measured using 25 previously developed items that have been reported to have content, concurrent, and construct validity.[21] These relate to five subscales, measured with five items each: 'Avoidant' (e.g. *"I postpone decision making whenever possible"*), 'dependent' (e.g. *"I rarely make important decisions without consulting other people"*), 'intuitive' (e.g. *"I generally make decisions that feel right to me"*), 'rational' (e.g. *"My decision-making requires careful thought"*), and 'spontaneous' (e.g. *"I make quick decisions"*). Item order was randomised for each participant. Reliability was good for all five subscales within the analysed sample (Cronbach's α: 0.71 to 0.81).

For peer review only - http://bmjopen.bmj.com/site/about/guidelines.xhtml

Overdiagnosis information: Text on overdiagnosis was followed by the question, *"How clear do you find this description of a risk of the test?"*. Response options were: *"Extremely clear"*, *"very clear"*, *"moderately clear"*, *"slightly clear"*, and *"not at all clear"*. Participants were also asked *"Have you ever read or heard similar information about a screening test?"* (response options: *"Yes"*, *"no"*, *"not sure"*).

Piloting

Piloting aimed to ensure that the survey was acceptable and manageable for participants, and that items were comprehensible. This consisted of a series of cognitive interviews[25] in which the survey was administered to 11 members of the general public via telephone and asked for feedback on whether any items were unclear, difficult to understand, or offensive, followed by a 'soft-launch' of the survey to 431 participants to ensure data were useable.

Analysis

Participants were excluded if data were missing, if they responded *"not sure"* or *"don't know"*, to any item, or had a non-ordinal level of education.

Items relating to each decision-making style were scored from one to five and summed to create an overall score (higher scores represented a greater tendency to use a given approach to decision-making) and highest level of education was categorised based on Levels 1-4 used by the Office of National Statistics.[26] Social class grade was categorised as *"Grades A or B"*, *"Grades C1 or C2"*, or *"Grades D or E"*. Ethnicity and marital status were dichotomised into *"White British"* vs. *"Other ethnic groups"* and *"Married or living as a couple"* vs. *"Single, widowed, divorced, or separated"*, respectively.

Descriptive statistics were used to summarise the analysed sample overall and within each response category of perceived clarity of the overdiagnosis information. Responses on PAPM items were also summarised (previous screening participation vs. no previous participation) separately for the four screening modalities for all individuals who completed

For peer review only - http://bmjopen.bmj.com/site/about/guidelines.xhtml

them (i.e. excluding those who were not eligible to be asked the question and those who declined to answer).

The main analysis tested the null hypothesis that overdiagnosis text (from breast vs. prostate information), demographics characteristics, previous exposure to screening information, and scores on the five DMS scales were not associated with the primary outcome (perceived clarity of information). The assumption of proportional odds was violated (test of parallel lines: p=.005), indicating that associations between independent variables and the outcome were not consistent for all possible ways of dichotomising perceived clarity (e.g. *"extremely"* or *"very clear"* vs. *"moderately"*, *"slightly"* or *"not at all clear"*). Hence, binary logistic regression was used instead of an ordinal logistic model, in which the outcome was dichotomised using the previously stated thresholds, since the authors considered this most conceptually meaningful. The authors made a collective judgement regarding the level of perceived clarity that should be considered appropriate but other thresholds can also be used. Table 1 and Table 2 illustrate the distribution of responses in all levels and Appendix 1 and Appendix 2 show the results of a sensitivity analysis in which the outcome is dichotomised at all other possible thresholds.

Demographic predictor variables used were gender, marital status, ethnicity, highest level of education, social class grade, previous diagnosis of cancer, and knowing someone diagnosed with cancer. Variables related to previous exposure to screening information consisted of having previously i) read a leaflet about screening, ii) read an NHS website about screening, iii) discussed screening with a doctor or nurse, and iv) read or heard similar information. Scores on the five DMS subscales were also included. The remaining predictor variable was text on overdiagnosis. There was minimal (multi)collinearity between predictor variables, meaning that associations between predictors and the outcome were unlikely to be notably affected by the inclusion or omission of other predictors (Variance Inflation Factors were ≤1.830). There were no violations of the assumption of linearity for continuous

For peer review only - http://bmjopen.bmj.com/site/about/guidelines.xhtml

independent variables (Box-Tidwell procedure: all p-values >.114), indicating that associations with the outcome were consistent across all values.

Adjusted odds-ratios (ORs) for rating the information as ‘extremely’ or ‘very clear’ vs. reporting a lower category of perceived clarity are reported with 95% confidence intervals (CIs) and p-values, alongside descriptive statistics.

RESULTS

Participant characteristics

Interviews were carried out with 2,111 participants; 495 were excluded due to missing or inapplicable data (Appendix 3), leaving a total of 1,616 in the main analysis. Percentages of participants who reported previously participating in screening were 75.2%, 80.5%, 60.9%, and 16.6% for cervical, breast, bowel, and prostate screening, respectively (n=732; n=343; n=407; n=349). Other sample characteristics are described in Table 1 and Table 2.

Predictors of rating the information on overdiagnosis as extremely/very clear

Participants were more likely to rate the breast overdiagnosis information as extremely or very clear (vs. moderately, slightly, or not at all clear) compared with the relevant information for prostate screening (OR: 1.43; 95% CI: 1.17 to 1.75; p=.001). Previous exposure to screening information was also associated: participants who had read a screening leaflet (1.35, 1.04 to 1.74; p=.024) or had read or heard similar information before (1.77, 1.40 to 2.23; p<.0005) were more likely to rate the information as extremely/very clear. However, previously reading an NHS website about screening (0.97, 0.72 to 1.30; p=.815) and talking to a health care professional about screening (1.08, 0.83 to 1.39; p=.576) was not associated (Table 1).

Rational decision-making style scores were associated: participants with higher scores were more likely to rate the information as extremely/very clear (1.06, 1.02 to 1.11; p=.009). There was moderate evidence against the null hypothesis for a relationship in the opposite

For peer review only - http://bmjopen.bmj.com/site/about/guidelines.xhtml

For peer review only

direction in the case of the dependent decision-making style (0.97, 0.97 to 1.00; p=.052). Trends are illustrated in Figure 1. There were no other associations between other variables and the outcome (Table 1 and Table 2).

DISCUSSION

In this survey of the general public in England, less than half of the sample rated either description of overdiagnosis as either extremely or very clear (38.4% and 46.8% for prostate and breast screening respectively). This is consistent with previous research reporting that lay people find the concept confusing and counterintuitive.[12,13] It also indicates that both pieces of information have room for improvement. To date, there has been little experimental research exploring the most effective methods of communicating overdiagnosis and related concepts in a way that would improve perceived clarity and potentially ameliorate decisional conflict;[15] previous research has assessed effects of manipulating disease terminology on treatment preferences (e.g. 'abnormal cells' as an alternative to 'ductal carcinoma in situ' in the case of breast cancer)[27] and effects of information formats on comprehension of overdiagnosis information in screening (text, fact boxes, and visual aids).[28] To our knowledge, this is the first study to compare how clear the public perceived two existing pieces of widely used information on overdiagnosis to be.

The information from the breast screening leaflet was somewhat more likely to be rated as clear than the equivalent information for prostate screening. This may be attributable to the intensive multistage development process that underpinned the revised breast information leaflet, consisting of a 'citizens' jury', expert input, cognitive testing with prospective service users, and combining feedback from experts and lay people. The main practical implication of this finding is that despite a (large) minority of participants rating the breast screening information as less than very clear, it may be more useful to adapt overdiagnosis information from this source when attempting to describe the concept in other contexts (rather than using the original information from prostate screening). For example, the NHS Abdominal

For peer review only - http://bmjopen.bmj.com/site/about/guidelines.xhtml

Aortic Aneurysm Programme also describes overdiagnosis with the following text: "…around 54 out of every 10,000 men screened will eventually have surgery to repair an aneurysm. On average, one of these 54 men will not survive the operation but their aneurysm may never have burst if left untreated".[29] It may be informative to assess how clear this information is perceived to be compared with or (given that the short length of this text means that there may be scope to develop the explanation further) in addition to information adapted from breast screening. It should also be noted that shortly before recruitment commenced on this study, Public Health England published a revised version of the prostate screening leaflet that included considerably more information on overdiagnosis (and overtreatment).[30] It would be valuable to determine how this new information compares to current information from breast screening (and AAA screening) as it may now be superior.

There were also associations between greater perceived clarity of the information and previous exposure to screening leaflets. Although unsurprising, this is reassuring in that it provides some evidence that the leaflets are providing enough background to be perceived as more clear at a later stage (48.6% of participants rated the information extremely/very clear among those who had read a leaflet vs 35.1% of those who had not). This was evident after controlling for exposure to similar information, (which was also associated) suggesting that it was not due to merely reencountering previously seen information.

Similar to our previous study on engagement with written screening information,[22] there was an association with a 'rational' decision-making style. Participants who were more inclined to make decisions in this way were more likely to find the information extremely or very clear, although it was surprising that this characteristic was associated with perceived clarity, whereas education was not. This finding suggests that the information is not serving individuals as well if they tend not to make decisions in this way. It may be beneficial to explore ways of describing the concept that require less deliberation, such as a more heavily summarised 'gist'[31] or an anecdotal 'narrative' approach, [32,33] describing a personal experience. There was also (weaker) evidence for a negative association between

For peer review only - http://bmjopen.bmj.com/site/about/guidelines.xhtml

dependent decision-making and perceived clarity. This may be mitigatable by providing clear instruction on who an individual can contact for further information (e.g. a primary care provider).

This study has limitations. First, generalisability of results may be reduced by the exclusion of participants with incomplete or inapplicable data. Second, although we added some background information regarding the aims and design of screening programmes, information on overdiagnosis was removed from its original context, which includes more detail on practicalities and outcomes of screening. If participants had encountered the information outside of a study context, perceived clarity may have been better (or possibly worse, in the presence of competing information points). The focus on perceived clarity of information meant that we did not measure other important variables such as objective understanding. Future research could quantify the extent to which the descriptions used are perceived as clear in more naturalistic contexts and whether they are understood correctly by more objective standards. Finally, the study aimed to determine predictors of perceived clarity in the general public, meaning that the sample was heterogeneous in terms of eligibility for specific screening modalities and the extent to which they were at risk of overdiagnosis in other healthcare contexts. In areas with a focus on communicating with a more specific group, it may be beneficial to test whether these findings replicate and whether other factors are relevant.

In conclusion, we found that information on overdiagnosis from the Breast Screening Programme in England is more likely to be rated as clearer than equivalent information from the Prostate Cancer Risk Management Programme and may be a valuable template for efforts to communicate the concept. Also, people who were less ‘rational’ and more ‘dependent’ in their decision-making may find existing information more challenging, suggesting that other descriptions may be appropriate for these individuals. Future research aiming to improve methods of communicating overdiagnosis information could assess

For peer review only - http://bmjopen.bmj.com/site/about/guidelines.xhtml

possible descriptions of overdiagnosis in terms of objective knowledge and test for associations within specific groups who may find the concept relevant.

Funding: The current study was supported by a programme grant from Cancer Research UK awarded to Prof Jane Wardle (C1418/A14134). Dr Jo Waller is supported by a Career Development Fellowship from Cancer Research UK (C7492/A17219). Cancer Research UK was not involved in the design of this study; the collection, analysis, or interpretation of the results; in the writing of the manuscript; or in the decision to submit for publication.

Competing interests: None declared.

Contributors: AG, CR, and JW conceived and designed the study. AG and EM analysed the data. AG, CR, and JW participated in the interpretation. All authors drafted the manuscript, participated in critical revision, and approved the final version.

Data sharing: No additional data are available.

REFERENCES

1 Glasziou P, Moynihan R, Richards T, Godlee F. Too much medicine; too little care. BMJ 2013;347:f4247.

2 McCaffery KJ, Jansen J, Scherer LD, et al. Walking the tightrope: communicating overdiagnosis in modern healthcare. BMJ 2016;352:i348.

3 Malhotra A, Maughan D, Ansell J, et al. Choosing Wisely in the UK: the Academy of Medical Royal Colleges' initiative to reduce the harms of too much medicine. BMJ 2015;350:h2308.

4 Journal of the American Medical Association. Less is More: http://jamanetwork.com/collections/6017/less-is-more Accessed: 28th November, 2016.

5 Calderwood C. Chief Medical Officer's annual report 2014-15. 2016.

For peer review only - http://bmjopen.bmj.com/site/about/guidelines.xhtml

http://www.gov.scot/Publications/2016/01/3745 Accessed: 28th November, 2016.

6 Hersch J, Barratt A, Jansen J, et al. Use of a decision aid including information on overdetection to support informed choice about breast cancer screening: a randomised controlled trial. Lancet 2015;385:1642-52.

7 Buchbinder R, Gross DP, Werner EL, Hayden JA. Understanding the characteristics of effective mass media campaigns for back pain and methodological challenges in evaluating their effects. Spine (Phila Pa 1976) 2008;33:74–80.

8 Moynihan R, Nickel B, Hersch J, et al. Public opinions about overdiagnosis: A national community survey. PLoS One 2015;10:e0125165.

9 Wegwarth O, Gigerenzer G. Less is more: Overdiagnosis and overtreatment: Evaluation of what physicians tell their patients about screening harms. JAMA Intern Med 2013;173:2086-7.

10 Schwartz LM, Woloshin S, Sox HC, Fischhoff B, Welch HG. US women's attitudes to false positive mammography results and detection of ductal carcinoma in situ: cross sectional survey. BMJ 2000;320:1635–40.

11 Ghanouni A, Meisel SF, Renzi C, Wardle J, Waller J. Public definitions of the term 'overdiagnosis' in the UK. BMJ Open 2016;6:e010723.

12 Hersch J, Jansen J, Barratt A, et al. Women's views on overdiagnosis in breast cancer screening: a qualitative study. BMJ 2013;346:f158.

13 Waller J, Douglas E, Whitaker KL, Wardle J. Women's responses to information about overdiagnosis in the UK breast cancer screening programme: a qualitative study. BMJ Open 2013;3:e002703.

14 Welch H, Schwartz L, Woloshin S. Overdiagnosed: making people sick in the pursuit of health. Beacon Press, 2011.

For peer review only - http://bmjopen.bmj.com/site/about/guidelines.xhtml

For peer review only

15 O'Connor AM. Validation of a decisional conflict scale. Medical Decision Making 1995;15(1):25-30.

16 House of Commons Science and Technology Committee. National Health Screening. Third Report of Session 2014-15. 2015. http://www.parliament.uk/business/committees/committees-a-z/commons-select/science-and-technology-committee/inquiries/parliament-2010/national-health-screening Accessed: 28th November, 2016.

17 Forbes LJ, Ramirez AJ, Expert group on Information about Breast Screening. Offering informed choice about breast screening. J Med Screen 2014;21:194–200.

18 National Breast Screening Programme. NHS Breast Screening: Helping you decide. 2013. https://www.gov.uk/government/publications/breast-screening-helping-women-decide Accessed: 28th November, 2016.

19 Public Health England. Prostate cancer risk management programme (PCRMP): benefits and risks of PSA testing. 2016. https://www.gov.uk/government/publications/prostate-cancer-risk-management-programme-psa-test-benefits-and-risks/prostate-cancer-risk-management-programme-pcrmp-benefits-and-risks-of-psa-testing Accessed: 28th November, 2016.

20 Burford D, Austoker J, Kirby M. PSA (prostate specific antigen) testing for prostate cancer. An information sheet for men considering a PSA test. 2009.

21 Scott SG, Bruce RA. Decision-making style: The development and assessment of a new measure. Educ Psychol Meas 1995;55:818–31.

22 Ghanouni A, Renzi C, Waller J. A cross-sectional survey assessing factors associated with reading cancer screening inormation: previous screening behaviour, demographics and decision-making style. BMC Public Health 2017;17:327.

For peer review only - http://bmjopen.bmj.com/site/about/guidelines.xhtml

23 National Readership Survey. Social grade. http://www.nrs.co.uk/nrs-print/lifestyle-and-classification-data/social-grade/ Accessed: 9th May 2017.

24 Weinstein ND. The precaution adoption process. Health Psychol 1988;7:355–86.

25 Willis GB, Artino AR. What do our respondents think we're asking? Using cognitive interviewing to improve medical education surveys. J Grad Med Educ 2013;5:353–6.

26 Office of National Statistics. 2011 Census: Key Statistics for England and Wales, March 2011. 2012. http://www.ons.gov.uk/ons/rel/census/2011-census/key-statistics-for-local-authorities-in-england-and-wales/stb-2011-census-key-statistics-for-england-and-wales.html Accessed: 28th November, 2016.

27 Nickel B, Barratt A, Hersch J, Moynihan R, Irwig L, McCaffery K. How different terminology for ductal carcinoma in situ (DCIS) impacts women's concern and management preferences: A qualitative study. Breast 2015;24:673–9.

28 Petrova D, Garcia-Retamero R, Cokely ET. Understanding the harms and benefits of cancer screening: a model of factors that shape informed decision making. Med Decis Making 2015;35:847-58.

29 Public Health England. Abdominal aortic aneurysm (AAA) screening, a free test for men aged 65 and over. 2015. https://www.gov.uk/government/publications/abdominal-aortic-aneurysm-screening-invitation-leaflet Accessed: 28th November, 2016.

30 Public Health England. PSA testing and prostate cancer: advice for well men aged 50 and over. 2016. https://www.gov.uk/government/publications/prostate-specific-antigen-testing-description-in-brief Accessed: 28th November, 2016.

31 Smith SG, Raine R, Obichere A, Wolf MS, Wardle J, von Wagner C. The effect of a supplementary ('gist-based') information leaflet on colorectal cancer knowledge and screening intention: a randomized controlled trial. J Behav Med 2015;38:261–72.

For peer review only - http://bmjopen.bmj.com/site/about/guidelines.xhtml

32 Green MC. Narratives and cancer communication. J Commun 2006;56:S163–83.

33 Hinyard LJ, Kreuter MW. Using narrative communication as a tool for health behavior change: a conceptual, theoretical, and empirical overview. Health Educ Behav 2007;34:777–92.

For peer review only

For peer review only - http://bmjopen.bmj.com/site/about/guidelines.xhtml

Table 1 – Perceived clarity of overdiagnosis information: descriptive statistics for categorical/ordinal variables, adjusted ORs, 95% CIs, p-values for variables in the multivariable binary logistic regression model

		"How clear do you find this description of a risk of the test?" n (%)					Adjusted OR, 95% CI; p-value
**Characteristic**	**Total (N=1,616)**	**Extremely clear (n=148; 9.2%)**	**Very clear (n=542; 33.5%)**	**Moderately clear (n=592; 36.6%)**	**Slightly clear (n=173; 10.7%)**	**Not at all clear (n=161; 10.0%)**	**Extremely/Very clear (vs. Not/Slightly/Moderately)**
**Overdiagnosis information**							
Breast screening text	850	75 (8.8)	321 (37.8)	285 (33.5)	81 (9.5)	88 (10.4)	1.43, 1.17 to 1.75; **.001**
vs. Prostate screening text	766	73 (9.5)	221 (28.9)	307 (40.1)	92 (12.0)	73 (9.5)	
**Gender**							
Male	748	66 (8.8)	228 (30.5)	303 (40.5)	81 (10.8)	70 (9.4)	0.92, 0.74 to 1.14; .447
vs. Female	868	82 (9.4)	314 (36.2)	289 (33.3)	92 (10.6)	91 (10.5)	
**Ethnicity**							
White British	1,229	116 (9.4)	425 (34.6)	447 (36.4)	121 (9.8)	120 (9.8)	1.19, 0.92 to 1.54; .197
vs. Other ethnic groups	387	32 (8.3)	117 (30.2)	145 (37.5)	52 (13.4)	41 (10.6)	
**Marital status**							
Married or living as a couple	987	92 (9.3)	344 (34.9)	355 (36)	101 (10.2)	95 (9.6)	1.16, 0.93 to 1.45; .184
vs. Single, widowed, divorced,	629	56 (8.9)	198 (31.5)	237 (37.7)	72 (11.4)	66 (10.5)	

For peer review only - http://bmjopen.bmj.com/site/about/guidelines.xhtml

or separated							
**Highest level of education**							Overall p-value: .482
No formal qualifications	275	24 (8.7)	97 (35.3)	107 (38.9)	25 (9.1)	22 (8.0)	1.24, 0.86 to 1.79; .248
Approximately Level 1, 2, or 3	858	68 (7.9)	297 (34.6)	312 (36.4)	97 (11.3)	84 (9.8)	1.13, 0.87 to 1.46; .353
vs. Approximately Level 4	483						
**Social class grade**							Overall p-value: .610
Grade A or B	351	41 (11.7)	117 (33.3)	122 (34.8)	31 (8.8)	40 (11.4)	1.18, 0.85 to 1.64; .323
Grade C1 or C2	786	71 (9.0)	267 (34.0)	298 (37.9)	80 (10.2)	70 (8.9)	1.09, 0.85 to 1.40; .507
vs. Grade D or E	479	36 (7.5)	158 (33.0)	172 (35.9)	62 (12.9)	51 (10.6)	
**Personal diagnosis of cancer**							
Yes	86	11 (12.8)	36 (41.9)	25 (29.1)	6 (7.0)	8 (9.3)	1.34, 0.84 to 2.12; .218
vs. No	1,530	137 (9.0)	506 (33.1)	567 (37.1)	167 (10.9)	153 (10.0)	
**Knows someone with cancer**							
Yes	946	89 (9.4)	348 (36.8)	313 (33.1)	97 (10.3)	99 (10.5)	1.23, 0.99 to 1.53; .060
vs. No	670	59 (8.8)	194 (29.0)	279 (41.6)	76 (11.3)	62 (9.3)	
**Previously read a screening leaflet**							
Yes	907	101 (11.1)	340 (37.5)	298 (32.9)	87 (9.6)	81 (8.9)	1.35, 1.04 to 1.74; **.024**
vs. No	709	47 (6.6)	202 (28.5)	294 (41.5)	86 (12.1)	80 (11.3)	

For peer review only - http://bmjopen.bmj.com/site/about/guidelines.xhtml

**Previously read an NHS screening website**							
Yes	275	33 (12.0)	105 (38.2)	88 (32.0)	23 (8.4)	26 (9.5)	0.97, 0.72 to 1.30; .815
vs. No	1,341	115 (8.6)	437 (32.6)	504 (37.6)	150 (11.2)	135 (10.1)	
**Discussed screening with doctor/nurse**							
Yes	535	67 (12.5)	200 (37.4)	172 (32.1)	46 (8.6)	50 (9.3)	1.08, 0.83 to 1.39; .576
vs. No	1,081	81 (7.5)	342 (31.6)	420 (38.9)	127 (11.7)	111 (10.3)	
**Previously read or heard similar information**							
Yes	584	78 (13.4)	239 (40.9)	189 (32.4)	49 (8.4)	29 (5.0)	1.77, 1.40 to 2.23; **<.0005**
vs. No	1,032	70 (6.8)	303 (29.4)	403 (39.1)	124 (12.0)	132 (12.8)	

N.B. Adjusted ORs and 95% CIs are relative to a stated reference category; p-values <.05 are in bold; all predictor variables are included in the model

For peer review only - http://bmjopen.bmj.com/site/about/guidelines.xhtml

Table 2 – Perceived clarity of overdiagnosis information: descriptive statistics for continuous variables, adjusted ORs, 95% CIs, p-values for variables in the multivariable binary logistic regression model

		"How clear do you find this description of a risk of the test?" M (SD)					Adjusted OR, 95% CI; p-value
**Characteristic**	**Total (n=1,616)**	**Extremely clear (n=148; 9.2%)**	**Very clear (n=542; 33.5%)**	**Moderately clear (n=592; 36.6%)**	**Slightly clear (n=173; 10.7%)**	**Not at all clear (n=161; 10.0%)**	**Extremely/Very clear (vs. Not/Slightly/Moderately)**
**Age (in years)**	43.7 (15.7)	45.4 (14.8)	44.3 (16)	43.3 (15.9)	43.2 (15.7)	42.4 (14.7)	1.00, 0.99 to 1.00; .282
**Decision-making styles**							
Avoidant score	13.8 (3.9)	13.3 (4.4)	13.8 (3.8)	13.9 (3.9)	13.7 (3.9)	13.5 (3.8)	1.01, 0.98 to 1.04; .713
Dependent score	16.9 (3.5)	16.2 (4)	16.8 (3.5)	17.0 (3.4)	17.4 (3.5)	16.5 (3.4)	0.97, 0.94 to 1.00; .052
Intuitive score	18.7 (2.8)	18.8 (3.4)	18.9 (2.7)	18.6 (2.7)	18.7 (2.7)	18.3 (2.6)	1.02, 0.98 to 1.06; .413
Rational score	19.7 (2.6)	20.4 (2.7)	19.7 (2.6)	19.6 (2.6)	19.7 (2.8)	19.4 (2.6)	1.06, 1.02 to 1.11; **.009**
Spontaneous score	14.7 (3.7)	14.5 (4.1)	14.9 (3.6)	14.9 (3.6)	14.5 (3.8)	14.0 (3.7)	1.03, 1.00 to 1.07; .073

N.B. Adjusted ORs and 95% CIs are per unit increase; p-values <.05 are in bold; all predictor variables are included in the model

For peer review only - http://bmjopen.bmj.com/site/about/guidelines.xhtml

Figure Legends:

Figure 1 – Proportion of participants who perceived the information as extremely/very clear within each possible score level for rational and dependent decision-making styles (percentage and total number of participants in brackets)

For peer review only

For peer review only - http://bmjopen.bmj.com/site/about/guidelines.xhtml

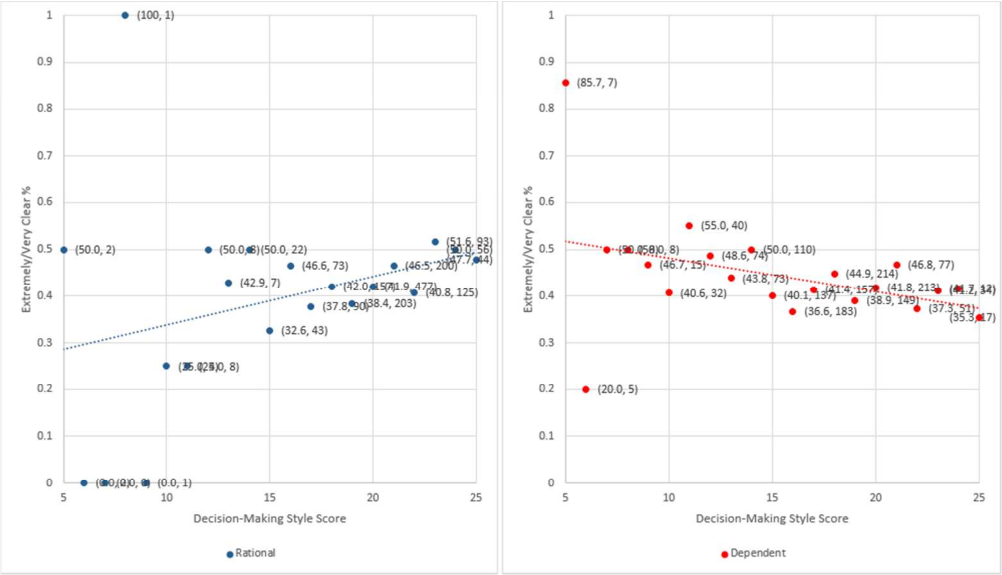

Figure 1 – Proportion of participants who perceived the information as extremely/very clear within each possible score level for rational and dependent decision-making styles (percentage and total number of participants in brackets)

84x48mm (300 x 300 DPI)

Appendix 1 – Sensitivity analyses: perceived clarity of overdiagnosis information: adjusted ORs, 95% CIs, p-values for categorical/ordinal variables in multivariable binary logistic regression models

	"How clear do you find this description of a risk of the test?" n (%)			
**Characteristic**	**Extremely (vs. less than extremely)**	**At least very (vs. less than very)**	**At least moderately (vs. less than moderately)**	**At least slightly (vs. not at all)**
	**Adjusted OR, 95% CI; p-value**	**Adjusted OR, 95% CI; p-value**	**Adjusted OR, 95% CI; p-value**	**Adjusted OR, 95% CI; p-value**
**Overdiagnosis information**				
Breast screening text vs. Prostate screening text	0.92, 0.65 to 1.30; .635	1.43, 1.17 to 1.75; **.001**	1.09, 0.85 to 1.40; .484	0.89, 0.64 to 1.24; .482
**Gender**				
Male vs. Female	1.03, 0.71 to 1.50; .873	0.92, 0.74 to 1.14; .447	1.20, 0.92 to 1.56; .183	1.24, 0.87 to 1.78; .243
**Ethnicity**				
White British vs. Other ethnic groups	1.26, 0.80 to 1.99; .312	1.19, 0.92 to 1.54; .197	1.35, 1.00 to 1.82; .054	1.17, 0.77 to 1.77; .464
**Marital status**				
Married or living as a couple vs. Single, widowed, divorced, or separated	0.98, 0.68 to 1.42; .909	1.16, 0.93 to 1.45; .184	1.12, 0.87 to 1.46; .382	1.03, 0.72 to 1.47; .879
**Highest level of education**	Overall: .535	Overall: .482	Overall: .075	Overall: .425

No formal qualifications	0.91, 0.50 to 1.66; .751	1.24, 0.86 to 1.79; .248	1.70, 1.07 to 2.68; .024	1.48, 0.80 to 2.75; .213
Approximately Level 1, 2, or 3	0.79, 0.52 to 1.20; .277	1.13, 0.87 to 1.46; .353	1.17, 0.86 to 1.59; .315	1.22, 0.82 to 1.84; .330
vs. Approximately Level 4				
**Social class grade**	Overall: .534	Overall: .610	Overall: .053	Overall: .260
Grade A or B	1.37, 0.79 to 2.38; .263	1.18, 0.85 to 1.64; .323	1.40, 0.95 to 2.07; .092	1.02, 0.61 to 1.70; .954
Grade C1 or C2	1.67, 0.75 to 1.83; .497	1.09, 0.85 to 1.40; .507	1.44, 1.07 to 1.94; .017	1.34, 0.89 to 2.02; .165
vs. Grade D or E				
**Personal diagnosis of cancer**				
Yes	1.23, 0.61 to 2.45; .565	1.34, 0.84 to 2.12; .218	1.20, 0.65 to 2.22; .557	1.03, 0.47 to 2.27; .934
vs. No				
**Knows someone with cancer**				
Yes	0.85, 0.59 to 1.23; .390	1.23, 0.99 to 1.53; .060	0.85, 0.65 to 1.11; .226	0.79, 0.55 to 1.13; .196
vs. No				
**Previously read a screening leaflet**				
Yes	1.30, 0.83 to 2.04; .257	1.35, 1.04 to 1.74; **.024**	1.12, 0.83 to 1.53; .461	1.11, 0.73 to 1.69; .619
vs. No				
**Previously read an NHS screening website**				
Yes	0.85, 0.54 to 1.36; .504	0.97, 0.72 to 1.30; .815	0.95, 0.65 to 1.39; .779	0.85, 0.51 to 1.42; .544
vs. No				

For peer review only

**Discussed screening with doctor/nurse**				
Yes	1.31, 0.87 to 1.97; .192	1.08, 0.83 to 1.39; .576	1.02, 0.74 to 1.41; .894	0.85, 0.55 to 1.30; .447
vs. No				
**Previously read or heard similar information**				
Yes	1.75, 1.19 to 2.57; **.004**	1.77, 1.40 to 2.23; **<.0005**	2.19, 1.61 to 2.98; **<.0005**	3.27, 2.08 to 5.14; **<.0005**
vs. No				

N.B. Adjusted ORs and 95% CIs are relative to a stated reference category; p-values <.05 are in bold; all predictor variables are included in each model with one exception (marked N/A in Appendix 2) due to a violation of the assumption linearity

For peer review only

Appendix 2 – Sensitivity analyses: perceived clarity of overdiagnosis information: adjusted ORs, 95% CIs, p-values for continuous variables in multivariable binary logistic regression models

	"How clear do you find this description of a risk of the test?" n (%)			
**Characteristic**	**Extremely (vs. less than extremely)**	**At least very (vs. less than very)**	**At least moderately (vs. less than moderately)**	**At least slightly (vs. not at all)**
	**Adjusted OR, 95% CI; p-value**	**Adjusted OR, 95% CI; p-value**	**Adjusted OR, 95% CI; p-value**	**Adjusted OR, 95% CI; p-value**
**Age (in years)**	1.00, 0.98 to 1.01; .642	1.00, 0.99 to 1.00; .282	1.00, 0.99 to 1.01; .329	1.00, 0.99 to 1.02; .562
**Decision-making styles**				
Avoidant score	1.01, 0.96 to 1.07; .711	1.01, 0.98 to 1.04; .713	1.03, 0.99 to 1.07; .143	0.99, 0.94 to 1.04; .611
Dependent score	0.94, 0.89 to 0.99; **.016**	0.97, 0.94 to 1.00; .052	0.98, 0.94 to 1.02; .289	1.04, 0.98 to 1.09; .176
Intuitive score	1.00, 0.94 to 1.07; .978	1.02, 0.98 to 1.06; .413	1.02, 0.98 to 1.07; .358	1.01, 0.94 to 1.07; .857
Rational score	1.15, 1.06 to 1.24; **.001**	1.06, 1.02 to 1.11; **.009**	1.03, 0.98 to 1.09; .215	1.08, 1.01 to 1.16; **.031**
Spontaneous score	1.02, 0.96 to 1.08; .574	1.03, 1.00 to 1.07; .073	N/A	1.09, 1.01 to 1.16; **.004**

N.B. Adjusted ORs and 95% CIs are per unit increase in the case of continuous variables; p-values <.05 are in bold; all predictor variables are included in each model with one exception (marked N/A) due to a violation of the assumption linearity

For peer review only

Appendix 3 - Numbers and percentages of participants excluded from the full sample of 2,111

**Excluded due to missing/inapplicable data on:**	**n (%)**
Reading a leaflet from the NHS about a screening test	39
Reading information about a screening test on an NHS website	15
Talking with a doctor or nurse about a screening test	18
Reading or hearing similar information about a screening test	74
Being diagnosed with cancer	20
Anyone close being diagnosed with cancer	13
Marital status	7
Education	216
Ethnicity	9
Perceived clarity of the description of a risk of the test	48
Decision-making style	36
**Total excluded**	**495 (23.4)**
**Total analysed**	**1,616**

For peer review only

STROBE Statement—checklist of items that should be included in reports of observational studies

	Item No	Recommendation	Checklist
**Title and abstract**	1	(*a*) Indicate the study's design with a commonly used term in the title or the abstract	P1-2
		(*b*) Provide in the abstract an informative and balanced summary of what was done and what was found	P2
**Introduction**			
Background/rationale	2	Explain the scientific background and rationale for the investigation being reported	P3-5
Objectives	3	State specific objectives, including any prespecified hypotheses	P4-5
**Methods**			
Study design	4	Present key elements of study design early in the paper	P5
Setting	5	Describe the setting, locations, and relevant dates, including periods of recruitment, exposure, follow-up, and data collection	P5-6
Participants	6	(*a*) *Cohort study*—Give the eligibility criteria, and the sources and methods of selection of participants. Describe methods of follow-up *Case-control study*—Give the eligibility criteria, and the sources and methods of case ascertainment and control selection. Give the rationale for the choice of cases and controls *Cross-sectional study*—Give the eligibility criteria, and the sources and methods of selection of participants	P5-6
		(*b*) *Cohort study*—For matched studies, give matching criteria and number of exposed and unexposed *Case-control study*—For matched studies, give matching criteria and the number of controls per case	N/A
Variables	7	Clearly define all outcomes, exposures, predictors, potential confounders, and effect modifiers. Give diagnostic criteria, if applicable	P9-10
Data sources/ measurement	8*	For each variable of interest, give sources of data and details of methods of assessment (measurement). Describe comparability of assessment methods if there is more than one group	P6-10
Bias	9	Describe any efforts to address potential sources of bias	P9-10
Study size	10	Explain how the study size was arrived at	P6
Quantitative variables	11	Explain how quantitative variables were handled in the analyses. If applicable, describe which groupings were chosen and why	P9-10
Statistical methods	12	(*a*) Describe all statistical methods, including those used to control for confounding	P9-10
		(*b*) Describe any methods used to examine subgroups and interactions	P9-10
		(*c*) Explain how missing data were addressed	P9
		(*d*) *Cohort study*—If applicable, explain how loss to follow-up was addressed *Case-control study*—If applicable, explain how matching of cases and controls was addressed *Cross-sectional study*—If applicable, describe analytical methods	P9-10

For peer review only - http://bmjopen.bmj.com/site/about/guidelines.xhtml

		taking account of sampling strategy	
		(*e*) Describe any sensitivity analyses	P10
**Results**			
Participants	13*	(a) Report numbers of individuals at each stage of study—eg numbers potentially eligible, examined for eligibility, confirmed eligible, included in the study, completing follow-up, and analysed	P11
		(b) Give reasons for non-participation at each stage	Appendix 3
		(c) Consider use of a flow diagram	N/A
Descriptive data	14*	(a) Give characteristics of study participants (eg demographic, clinical, social) and information on exposures and potential confounders	P11; Table 1; Table 2
		(b) Indicate number of participants with missing data for each variable of interest	Appendix 3
		(c) *Cohort study*—Summarise follow-up time (eg, average and total amount)	N/A
Outcome data	15*	*Cohort study*—Report numbers of outcome events or summary measures over time	P11; Table 1; Table 2
		Case-control study—Report numbers in each exposure category, or summary measures of exposure	
		Cross-sectional study—Report numbers of outcome events or summary measures	
Main results	16	(*a*) Give unadjusted estimates and, if applicable, confounder-adjusted estimates and their precision (eg, 95% confidence interval). Make clear which confounders were adjusted for and why they were included	P11; Table 1; Table 2
		(*b*) Report category boundaries when continuous variables were categorized	Table 1; Table 2
		(*c*) If relevant, consider translating estimates of relative risk into absolute risk for a meaningful time period	N/A
Other analyses	17	Report other analyses done—eg analyses of subgroups and interactions, and sensitivity analyses	Appendix 1 Appendix 2
**Discussion**			
Key results	18	Summarise key results with reference to study objectives	P12-13
Limitations	19	Discuss limitations of the study, taking into account sources of potential bias or imprecision. Discuss both direction and magnitude of any potential bias	P14
Interpretation	20	Give a cautious overall interpretation of results considering objectives, limitations, multiplicity of analyses, results from similar studies, and other relevant evidence	P12-15
Generalisability	21	Discuss the generalisability (external validity) of the study results	P13-15
**Other information**			
Funding	22	Give the source of funding and the role of the funders for the present study and, if applicable, for the original study on which the present article is based	P15

*Give information separately for cases and controls in case-control studies and, if applicable, for exposed and unexposed groups in cohort and cross-sectional studies.

For peer review only - http://bmjopen.bmj.com/site/about/guidelines.xhtml

Note: An Explanation and Elaboration article discusses each checklist item and gives methodological background and published examples of transparent reporting. The STROBE checklist is best used in conjunction with this article (freely available on the Web sites of PLoS Medicine at http://www.plosmedicine.org/, Annals of Internal Medicine at http://www.annals.org/, and Epidemiology at http://www.epidem.com/). Information on the STROBE Initiative is available at www.strobe-statement.org.

For peer review only

For peer review only - http://bmjopen.bmj.com/site/about/guidelines.xhtml

BMJ Open

Comparing perceived clarity of information on overdiagnosis used for breast and prostate cancer screening in England: An experimental survey

Journal:	*BMJ Open*
Manuscript ID	bmjopen-2017-015955.R2
Article Type:	Research
Date Submitted by the Author:	16-Jun-2017
Complete List of Authors:	Ghanouni, Alex; University College London, Research Department of Behavioural Science and Health<br>Renzi, Cristina; UCL, Research Department of Behavioural Science and Health<br>McBride, Emily; University College London, Research Department of Behavioural Science and Health<br>Waller, Jo; UCL, Research Department of Behavioural Science and Health
**Primary Subject Heading**:	Communication
Secondary Subject Heading:	Public health
Keywords:	Decision Making, Surveys and Questionnaires, Early Detection of Cancer, Mass Screening, Medical Overuse

SCHOLARONE™ Manuscripts

For peer review only

FULL TITLE PAGE

Title: Comparing perceived clarity of information on overdiagnosis used for breast and prostate cancer screening in England: An experimental survey

Manuscript type: Research article

Authors:

Alex Ghanouni[a] a.ghanouni@ucl.ac.uk

Cristina Renzi[a] c.renzi@ucl.ac.uk

Emily McBride[a] e.mcbride@ucl.ac.uk

Jo Waller[ab] j.waller@ucl.ac.uk | Tel: +44 (0)20 7679 5958 | Fax: +44 (0)20 7679 8354

Affiliations:

[a]Research Department of Behavioural Science and Health, University College London, Gower Street, London, WC1E 6BT, United Kingdom

[b]Corresponding author

Word count: Abstract: 300/300; Body: 3,560/4,000

For peer review only

For peer review only - http://bmjopen.bmj.com/site/about/guidelines.xhtml

ABSTRACT

Objectives: 'Overdiagnosis', detection of disease that would never have caused symptoms or death, is a public health concern due to possible psychological and physical harm but little is known about how best to explain it. This study evaluated public perceptions of widely-used information on the concept to identify scope for improving communication methods.

Design: Experimental survey carried out by a market research company via face-to-face computer-assisted interviews.

Setting: Interviews took place in participants' homes.

Participants: 2,111 members of the general public in England aged 18-70 years began the survey; 1,616 were eligible for analysis. National representativeness was sought via demographic quota sampling.

Interventions: Participants were allocated at random to receive a brief description of overdiagnosis derived from written information used by either the NHS Breast Screening Programme or the prostate cancer screening equivalent.

Primary and secondary outcome measures: The primary outcome was how clear the information was perceived to be (extremely/very clear vs. less clear). Other measures included previous exposure to screening information, decision-making styles, and demographic characteristics (e.g. education). Binary logistic regression was used to assess predictors of perceived clarity.

Results: Overdiagnosis information from the BSP was more likely to be rated as more clear compared with the prostate screening equivalent (adjusted odds-ratio: 1.43, 95% confidence interval: 1.17 to 1.75; p=.001). Participants were more likely to perceive the information as more clear if they had previously encountered similar information (OR: 1.77, 1.40 to 2.23; p<.0005) or a screening leaflet (OR: 1.35, 1.04 to 1.74; p=.024), or had a more 'rational' decision-making style (OR: 1.06, 1.02 to 1.11; p=.009).

For peer review only - http://bmjopen.bmj.com/site/about/guidelines.xhtml

Conclusions: Overdiagnosis information from breast screening may be a useful template for communicating the concept more generally (e.g. via organised campaigns). However, this information may be less well-suited to individuals who are less inclined to consider risks and benefits during decision-making.

Keywords: Decision Making; Surveys and Questionnaires; Early Detection of Cancer; Mass Screening; Medical Overuse

For peer review only

Strengths and limitations of this study:

- There has been very little research regarding how to effectively communicate the concept of overdiagnosis to lay people, which this study aims to begin to address
- It benefited from measuring a wide range of predictor variables in a large sample of the general public in which quotas were set with the aim of achieving population-representativeness
- The sample was also heterogeneous, meaning that findings may not apply equally to a specific healthcare context in which overdiagnosis may be an issue
- Information on overdiagnosis was extracted from information sources and presented separately, meaning that perceived clarity may be different when read within the original contexts
- Participants' objective understanding of the information was not measured and it is unknown how well this correlates with perceived clarity

INTRODUCTION

Overdiagnosis, defined as the detection of disease that would never have caused symptoms or death within a person's lifetime, is a topic of widespread concern in healthcare[1] since it results in negative psychological consequences, overtreatment, and overutilisation of scarce resources.[2] This has led to various efforts to communicate the issue to patients and the

For peer review only - http://bmjopen.bmj.com/site/about/guidelines.xhtml

public, increasing awareness so that it can be factored into decision-making around health.[1,3–7]

However, these efforts have proved challenging. For example, there is strong, consistent evidence that public awareness of overdiagnosis remains markedly low, both in the UK and internationally.[8–10] Fewer than one in three individuals surveyed in the UK reported recognising the term and virtually no participants were able to define it accurately.[11] This demonstrates that there is considerable scope for improving understanding of the concept, although research into effective communication methods is limited.[2] The available studies have also shown that the concept is difficult to explain in a way that lay people find clear. The term is often considered counterintuitive[12,13] since it contradicts an established faith in the importance of early detection.[14] A perceived lack of clarity of information on overdiagnosis is problematic since it is likely to result in feelings of uncertainty, contributing to decisional conflict.[15]

Breast and prostate cancer screening are two notable contexts in which overdiagnosis is a prominent issue.[16] In the UK, a thorough review of written information materials was carried out for the Breast Screening Programme with the explicit aim of improving communication of the concept.[17,18] Similarly, men receiving care under the Prostate Cancer Risk Management Programme are informed about overdiagnosis, primarily by clinicians but also via associated leaflets.[19,20] These written materials offer two brief descriptions of the concept that could potentially serve as models for communicating it more generally (e.g. to screening invitees or the wider public).

Strategies for communicating overdiagnosis may also become apparent via a better understanding of factors associated with perceived clarity of explanations, such as people's approaches to making important decisions ('decision-making styles'; DMS).[21] We previously explored whether this was associated with the amount of cancer screening information leaflets that screening-eligible individuals had read; less 'rational' participants who tended

For peer review only - http://bmjopen.bmj.com/site/about/guidelines.xhtml

not to weigh up available information were less likely to have read information in leaflets used by bowel and cervical screening programmes.[22] Since existing written information has been designed to allow individuals to consider risks and benefits, it is plausible that people who are less inclined to make decisions in this way would find materials less clear. There may also be relevant associations with other DMS (e.g. 'avoidant' or 'dependent' styles) or demographic characteristics (e.g. education and previous exposure to screening information).

We carried out a large-scale experimental survey of the general public in England to assess perceived clarity of existing, widely-used information on overdiagnosis derived from written material for breast and prostate cancer screening. We compared perceived clarity of these two pieces of information and tested for associated characteristics (specifically, DMS, demographics, and previous exposure to screening information).

METHOD

Design

Institutional ethical approval was obtained from the University College London Research Ethics Committee (5771/002). The study design and some aspects of the measures have also been described elsewhere.[22] Data were collected between April and May, 2016, as part of Wave 3 of the Attitudes, Behaviour and Cancer UK Survey (ABACUS). A market research agency (TNS) conducted face-to-face computer-assisted interviews in people's homes, as part of their weekly omnibus survey in England. Participants were presented with information describing overdiagnosis from either the leaflet used by the NHS Breast Screening Programme or the equivalent used by the Prostate Cancer Risk Management Programme in England, allocated at random in a 1:1 ratio. Participants were told:

"We would now like to ask you some questions about leaflets on health-related topics. The NHS offers people a variety of screening tests to check for illnesses before symptoms have appeared. People offered an NHS screening test are often given a leaflet that explains the

For peer review only - http://bmjopen.bmj.com/site/about/guidelines.xhtml

risks and benefits of having the test. The leaflet is either posted or given out by a doctor or nurse."

After completing items on previous exposure to screening information (described below), participants were given one of the following pieces of information:

"The test can find an illness that would never have caused a person harm. Some people will be diagnosed and treated for an illness that would never otherwise have been found and would not have become life-threatening." (From the breast screening information leaflet)[18]

"The test may make you worry by finding an illness that may never cause any symptoms or shorten your life." (From the prostate screening information sheet)[20]

The provided descriptions consisted of all the text relating to overdiagnosis in the prostate screening leaflet (as it existed at the time the survey was designed) and all relevant conceptual information from the breast screening leaflet (i.e. omitting overlapping but context-specific information on ductal carcinoma in situ and ratios of breast cancer deaths prevented to overdiagnosed cases). Text that reiterated the information above with slightly different wording was also omitted. The breast screening leaflet is freely available online; the original prostate screening leaflet is available on request.

Participants

Random location sampling was conducted using Census statistics and the Postcode Address File. National representativeness was achieved via quotas for demographic characteristics (e.g. employment status and gender). Participants were eligible if they were aged between 18 and 70 years. The total number of participants approached for this study was determined by funding constraints for the broader ABACUS survey.

Measures

For peer review only - http://bmjopen.bmj.com/site/about/guidelines.xhtml

Demographics: Principal measures were gender, ethnicity, marital status, highest level of education qualification obtained, and age. We also measured social class grade (a widely used classification of socioeconomic status based on type of employment).[23] Participants also stated whether they had been diagnosed with cancer (and what type, if applicable), or if they knew anyone who had been diagnosed.

Previous exposure to screening information: Participants were asked three questions:

1) *"Have you ever read a leaflet from the NHS about a screening test?"*

2) *"Have you ever read information about a screening test on an NHS website?"*

3) *"Have you ever talked with a doctor or nurse about a screening test?"*

(Response options: *"Yes"*, *"no"*, *"not sure"*).

If eligible for either cervical, breast, or bowel screening programmes in England and anticipated not to find the question distressing, participants were asked to state their previous screening history using up to four items based on the Precaution Adoption Process Model (PAPM)[24] e.g.:

"The next set of questions are about breast screening. The NHS breast screening programme invites women to have regular mammograms (x-rays of their breasts). "Which one of the following best describes you?"

Response options were: *"I have never heard of breast screening, I have heard of breast screening but have never been invited, I have been invited to breast screening but have never been, I have been invited to breast screening but have not been every time I was invited, I have been invited to breast screening and have been every time I was invited"*

Since prostate screening in England is carried out via direct contact with clinicians rather than an organised programme, previous participation was assessed in the following way among eligible participants:

For peer review only - http://bmjopen.bmj.com/site/about/guidelines.xhtml

"Men can have a blood test, called a prostate-specific antigen (PSA) test, to look for a protein in their blood that can be an indicator of prostate cancer. Which of the following best describes you?"

Response options were: *"I have never heard of a PSA test, I have heard of a PSA test but have never discussed it with a doctor or nurse, I have discussed a PSA test with a doctor or nurse but have never had it, I have discussed a PSA test with a doctor or nurse and have had the test, I have had the PSA test but I have never discussed it with a doctor or nurse"*

Participants were not asked these questions if they had previously been diagnosed with the applicable type of cancer, or if they were ineligible in terms of their gender or age (i.e. not aged 18-64 years for cervical screening questions, 47-70 years for breast screening questions, 58-70 for bowel screening questions, or 45-70 years for prostate screening questions).

Decision-making styles: Participants were informed:

"*We would like to start by asking you some questions about how you make important decisions. I am going to read out some statements describing how individuals go about making important decisions and I would like you to tell me how much you agree or disagree with each one."*

Tendencies towards using each decision-making style were measured using 25 previously developed items that have been reported to have content, concurrent, and construct validity.[21] These relate to five subscales, measured with five items each: 'Avoidant' (e.g. *"I postpone decision making whenever possible"*), 'dependent' (e.g. *"I rarely make important decisions without consulting other people"*), 'intuitive' (e.g. *"I generally make decisions that feel right to me"*), 'rational' (e.g. *"My decision-making requires careful thought"*), and 'spontaneous' (e.g. *"I make quick decisions"*). Item order was randomised for each participant. Reliability was good for all five subscales within the analysed sample (Cronbach's α: 0.71 to 0.81).

For peer review only - http://bmjopen.bmj.com/site/about/guidelines.xhtml

Overdiagnosis information: Text on overdiagnosis was followed by the question, *"How clear do you find this description of a risk of the test?"*. Response options were: *"Extremely clear"*, *"very clear"*, *"moderately clear"*, *"slightly clear"*, and *"not at all clear"*. Participants were also asked *"Have you ever read or heard similar information about a screening test?"* (response options: *"Yes"*, *"no"*, *"not sure"*).

Piloting

Piloting aimed to ensure that the survey was acceptable and manageable for participants, and that items were comprehensible. This consisted of a series of cognitive interviews[25] in which the survey was administered to 11 members of the general public via telephone and asked for feedback on whether any items were unclear, difficult to understand, or offensive, followed by a 'soft-launch' of the survey to 431 participants to ensure data were useable.

Analysis

Participants were excluded if data were missing, if they responded *"not sure"* or *"don't know"*, to any item, or had a non-ordinal level of education.

Items relating to each decision-making style were scored from one to five and summed to create an overall score (higher scores represented a greater tendency to use a given approach to decision-making) and highest level of education was categorised based on Levels 1-4 used by the Office of National Statistics.[26] Social class grade was categorised as *"Grades A or B"*, *"Grades C1 or C2"*, or *"Grades D or E"*. Ethnicity and marital status were dichotomised into *"White British"* vs. *"Other ethnic groups"* and *"Married or living as a couple"* vs. *"Single, widowed, divorced, or separated"*, respectively.

Descriptive statistics were used to summarise the analysed sample overall and within each response category of perceived clarity of the overdiagnosis information. Responses on PAPM items were also summarised (previous screening participation vs. no previous participation) separately for the four screening modalities for all individuals who completed

For peer review only - http://bmjopen.bmj.com/site/about/guidelines.xhtml

For peer review only

them (i.e. excluding those who were not eligible to be asked the question and those who declined to answer).

The main analysis tested the null hypothesis that overdiagnosis text (from breast vs. prostate information), demographics characteristics, previous exposure to screening information, and scores on the five DMS scales were not associated with the primary outcome (perceived clarity of information). The assumption of proportional odds was violated (test of parallel lines: p=.005), indicating that associations between independent variables and the outcome were not consistent for all possible ways of dichotomising perceived clarity (e.g. *"extremely"* or *"very clear"* vs. *"moderately"*, *"slightly"* or *"not at all clear"*). Hence, binary logistic regression was used instead of an ordinal logistic model, in which the outcome was dichotomised using the previously stated thresholds, since the authors considered this most conceptually meaningful. The authors made a collective judgement regarding the level of perceived clarity that should be considered appropriate but other thresholds can also be used. Table 1 and Table 2 illustrate the distribution of responses in all levels and Appendix 1 and Appendix 2 show the results of a sensitivity analysis in which the outcome is dichotomised at all other possible thresholds.

Demographic predictor variables used were gender, marital status, ethnicity, highest level of education, social class grade, previous diagnosis of cancer, and knowing someone diagnosed with cancer. Variables related to previous exposure to screening information consisted of having previously i) read a leaflet about screening, ii) read an NHS website about screening, iii) discussed screening with a doctor or nurse, and iv) read or heard similar information. Scores on the five DMS subscales were also included. The remaining predictor variable was text on overdiagnosis. There was minimal (multi)collinearity between predictor variables, meaning that associations between predictors and the outcome were unlikely to be notably affected by the inclusion or omission of other predictors (Variance Inflation Factors were ≤1.830). There were no violations of the assumption of linearity for continuous

For peer review only - http://bmjopen.bmj.com/site/about/guidelines.xhtml

independent variables (Box-Tidwell procedure: all p-values >.114), indicating that associations with the outcome were consistent across all values.

Adjusted odds-ratios (ORs) for rating the information as ‘extremely’ or ‘very clear’ vs. reporting a lower category of perceived clarity are reported with 95% confidence intervals (CIs) and p-values, alongside descriptive statistics.

RESULTS

Participant characteristics

Interviews were carried out with 2,111 participants; 495 were excluded due to missing or inapplicable data (Appendix 3), leaving a total of 1,616 in the main analysis. Percentages of participants who reported previously participating in screening were 75.2%, 80.5%, 60.9%, and 16.6% for cervical, breast, bowel, and prostate screening, respectively (n=732; n=343; n=407; n=349). Other sample characteristics are described in Table 1 and Table 2.

Predictors of rating the information on overdiagnosis as extremely/very clear

Participants were more likely to rate the breast overdiagnosis information as extremely or very clear (vs. moderately, slightly, or not at all clear) compared with the relevant information for prostate screening (OR: 1.43; 95% CI: 1.17 to 1.75; p=.001). Previous exposure to screening information was also associated: participants who had read or heard similar information before (1.77, 1.40 to 2.23; p<.0005) or had read a screening leaflet (1.35, 1.04 to 1.74; p=.024) were more likely to rate the information as extremely/very clear. However, previously reading an NHS website about screening (0.97, 0.72 to 1.30; p=.815) and talking to a health care professional about screening (1.08, 0.83 to 1.39; p=.576) was not associated (Table 1).

Rational decision-making style scores were associated: participants with higher scores were more likely to rate the information as extremely/very clear (1.06, 1.02 to 1.11; p=.009). There was moderate evidence against the null hypothesis for a relationship in the opposite

For peer review only - http://bmjopen.bmj.com/site/about/guidelines.xhtml

direction in the case of the dependent decision-making style (0.97, 0.97 to 1.00; p=.052). Trends are illustrated in Figure 1. There were no other associations between other variables and the outcome (Table 1 and Table 2).

DISCUSSION

In this survey of the general public in England, less than half of the sample rated either description of overdiagnosis as either extremely or very clear (38.4% and 46.8% for prostate and breast screening respectively). This is consistent with previous research reporting that lay people find the concept confusing and counterintuitive.[12,13] It also indicates that both pieces of information have room for improvement. To date, there has been little experimental research exploring the most effective methods of communicating overdiagnosis and related concepts in a way that would improve perceived clarity and potentially ameliorate decisional conflict;[15] previous research has assessed effects of manipulating disease terminology on treatment preferences (e.g. 'abnormal cells' as an alternative to 'ductal carcinoma in situ' in the case of breast cancer)[27] and effects of information formats on comprehension of overdiagnosis information in screening (text, fact boxes, and visual aids).[28] To our knowledge, this is the first study to compare how clear the public perceived two existing pieces of widely used information on overdiagnosis to be.

The information from the breast screening leaflet was somewhat more likely to be rated as clear than the equivalent information for prostate screening. This may be attributable to the intensive multistage development process that underpinned the revised breast information leaflet, consisting of a 'citizens' jury', expert input, cognitive testing with prospective service users, and combining feedback from experts and lay people. The main practical implication of this finding is that despite a (large) minority of participants rating the breast screening information as less than very clear, it may be more useful to adapt overdiagnosis information from this source when attempting to describe the concept in other contexts (rather than using the original information from prostate screening). For example, the NHS Abdominal

For peer review only - http://bmjopen.bmj.com/site/about/guidelines.xhtml

Aortic Aneurysm Programme also describes overdiagnosis with the following text: "…around 54 out of every 10,000 men screened will eventually have surgery to repair an aneurysm. On average, one of these 54 men will not survive the operation but their aneurysm may never have burst if left untreated".[29] It may be informative to assess how clear this information is perceived to be compared with or (given that the short length of this text means that there may be scope to develop the explanation further) in addition to information adapted from breast screening. It should also be noted that shortly before recruitment commenced on this study, Public Health England published a revised version of the prostate screening leaflet that included considerably more information on overdiagnosis (and overtreatment).[30] It would be valuable to determine how this new information compares to current information from breast screening (and AAA screening) as it may now be superior.

There was also an association between greater perceived clarity and having previously encountered similar information; the odds ratio was notably high for this variable compared to that of others. To the extent that this represents a causal relationship, this finding suggests that perceived clarity is limited by a general lack of familiarity with the concept. This supports the rationale for ongoing communication campaigns,[1,3-5] in which one of their aims is to create more instances in which people are exposed to information about overdiagnosis. A similar association was observed with previously reading a screening leaflet: Although unsurprising, this is reassuring in that it provides some evidence that the leaflets are providing enough background to be perceived as more clear at a later stage (48.6% of participants rated the information extremely/very clear among those who had read a leaflet vs 35.1% of those who had not). This was evident after controlling for exposure to similar information, (which was also associated) suggesting there was a unique effect beyond merely reencountering previously seen information.

Similar to our previous study on engagement with written screening information,[22] there was an association with a 'rational' decision-making style. Participants who were more inclined to make decisions in this way were more likely to find the information extremely or very clear,

For peer review only - http://bmjopen.bmj.com/site/about/guidelines.xhtml

although it was surprising that this characteristic was associated with perceived clarity, whereas education was not. This finding suggests that the information is not serving individuals as well if they tend not to make decisions in this way. It may be beneficial to explore ways of describing the concept that require less deliberation, such as a more heavily summarised 'gist'[31] or an anecdotal 'narrative' approach, [32,33] describing a personal experience. There was also (weaker) evidence for a negative association between dependent decision-making and perceived clarity. This may be mitigatable by providing clear instruction on who an individual can contact for further information (e.g. a primary care provider).

This study has limitations. First, generalisability of results may be reduced by the exclusion of participants with incomplete or inapplicable data. Second, although we added some background information regarding the aims and design of screening programmes, information on overdiagnosis was removed from its original context, which includes more detail on practicalities and outcomes of screening. If participants had encountered the information outside of a study context, perceived clarity may have been better (or possibly worse, in the presence of competing information points). The focus on perceived clarity of information meant that we did not measure other important variables such as objective understanding. Future research could quantify the extent to which the descriptions used are perceived as clear in more naturalistic contexts and whether they are understood correctly by more objective standards. Finally, the study aimed to determine predictors of perceived clarity in the general public, meaning that the sample was heterogeneous in terms of eligibility for specific screening modalities and the extent to which they were at risk of overdiagnosis in other healthcare contexts. In areas with a focus on communicating with a more specific group, it may be beneficial to test whether these findings replicate and whether other factors are relevant.

In conclusion, we found that information on overdiagnosis from the Breast Screening Programme in England is more likely to be rated as clearer than equivalent information from

For peer review only - http://bmjopen.bmj.com/site/about/guidelines.xhtml

the Prostate Cancer Risk Management Programme and may be a valuable template for efforts to communicate the concept. Also, people who were less 'rational' and more 'dependent' in their decision-making may find existing information more challenging, suggesting that other descriptions may be appropriate for these individuals. Future research aiming to improve methods of communicating overdiagnosis information could assess possible descriptions of overdiagnosis in terms of objective knowledge and test for associations within specific groups who may find the concept relevant. These findings provide some evidence that communication can be improved by creating additional opportunities for people to engage with information about the concept (e.g. via organised campaigns).

Funding: The current study was supported by a programme grant from Cancer Research UK awarded to Prof Jane Wardle (C1418/A14134). Dr Jo Waller is supported by a Career Development Fellowship from Cancer Research UK (C7492/A17219). Cancer Research UK was not involved in the design of this study; the collection, analysis, or interpretation of the results; in the writing of the manuscript; or in the decision to submit for publication.

Competing interests: None declared.

Contributors: AG, CR, and JW conceived and designed the study. AG and EM analysed the data. AG, CR, and JW participated in the interpretation. All authors drafted the manuscript, participated in critical revision, and approved the final version.

Data sharing: No additional data are available.

REFERENCES

1 Glasziou P, Moynihan R, Richards T, Godlee F. Too much medicine; too little care. BMJ 2013;347:f4247.

2 McCaffery KJ, Jansen J, Scherer LD, et al. Walking the tightrope: communicating overdiagnosis in modern healthcare. BMJ 2016;352:i348.

For peer review only - http://bmjopen.bmj.com/site/about/guidelines.xhtml

3 Malhotra A, Maughan D, Ansell J, et al. Choosing Wisely in the UK: the Academy of Medical Royal Colleges' initiative to reduce the harms of too much medicine. BMJ 2015;350:h2308.

4 Journal of the American Medical Association. Less is More: http://jamanetwork.com/collections/6017/less-is-more Accessed: 28th November, 2016.

5 Calderwood C. Chief Medical Officer's annual report 2014-15. 2016. http://www.gov.scot/Publications/2016/01/3745 Accessed: 28th November, 2016.

6 Hersch J, Barratt A, Jansen J, et al. Use of a decision aid including information on overdetection to support informed choice about breast cancer screening: a randomised controlled trial. Lancet 2015;385:1642-52.

7 Buchbinder R, Gross DP, Werner EL, Hayden JA. Understanding the characteristics of effective mass media campaigns for back pain and methodological challenges in evaluating their effects. Spine (Phila Pa 1976) 2008;33:74–80.

8 Moynihan R, Nickel B, Hersch J, et al. Public opinions about overdiagnosis: A national community survey. PLoS One 2015;10:e0125165.

9 Wegwarth O, Gigerenzer G. Less is more: Overdiagnosis and overtreatment: Evaluation of what physicians tell their patients about screening harms. JAMA Intern Med 2013;173:2086-7.

10 Schwartz LM, Woloshin S, Sox HC, Fischhoff B, Welch HG. US women's attitudes to false positive mammography results and detection of ductal carcinoma in situ: cross sectional survey. BMJ 2000;320:1635–40.

11 Ghanouni A, Meisel SF, Renzi C, Wardle J, Waller J. Public definitions of the term 'overdiagnosis' in the UK. BMJ Open 2016;6:e010723.

For peer review only - http://bmjopen.bmj.com/site/about/guidelines.xhtml

12 Hersch J, Jansen J, Barratt A, et al. Women's views on overdiagnosis in breast cancer screening: a qualitative study. BMJ 2013;346:f158.

13 Waller J, Douglas E, Whitaker KL, Wardle J. Women's responses to information about overdiagnosis in the UK breast cancer screening programme: a qualitative study. BMJ Open 2013;3:e002703.

14 Welch H, Schwartz L, Woloshin S. Overdiagnosed: making people sick in the pursuit of health. Beacon Press, 2011.

15 O'Connor AM. Validation of a decisional conflict scale. Medical Decision Making 1995;15(1):25-30.

16 House of Commons Science and Technology Committee. National Health Screening. Third Report of Session 2014-15. 2015. http://www.parliament.uk/business/committees/committees-a-z/commons-select/science-and-technology-committee/inquiries/parliament-2010/national-health-screening Accessed: 28th November, 2016.

17 Forbes LJ, Ramirez AJ, Expert group on Information about Breast Screening. Offering informed choice about breast screening. J Med Screen 2014;21:194–200.

18 National Breast Screening Programme. NHS Breast Screening: Helping you decide. 2013. https://www.gov.uk/government/publications/breast-screening-helping-women-decide Accessed: 28th November, 2016.

19 Public Health England. Prostate cancer risk management programme (PCRMP): benefits and risks of PSA testing. 2016. https://www.gov.uk/government/publications/prostate-cancer-risk-management-programme-psa-test-benefits-and-risks/prostate-cancer-risk-management-programme-pcrmp-benefits-and-risks-of-psa-testing Accessed: 28th November, 2016.

For peer review only - http://bmjopen.bmj.com/site/about/guidelines.xhtml

20 Burford D, Austoker J, Kirby M. PSA (prostate specific antigen) testing for prostate cancer. An information sheet for men considering a PSA test. 2009.

21 Scott SG, Bruce RA. Decision-making style: The development and assessment of a new measure. Educ Psychol Meas 1995;55:818–31.

22 Ghanouni A, Renzi C, Waller J. A cross-sectional survey assessing factors associated with reading cancer screening inormation: previous screening behaviour, demographics and decision-making style. BMC Public Health 2017;17:327.

23 National Readership Survey. Social grade. http://www.nrs.co.uk/nrs-print/lifestyle-and-classification-data/social-grade/ Accessed: 9th May 2017.

24 Weinstein ND. The precaution adoption process. Health Psychol 1988;7:355–86.

25 Willis GB, Artino AR. What do our respondents think we're asking? Using cognitive interviewing to improve medical education surveys. J Grad Med Educ 2013;5:353–6.

26 Office of National Statistics. 2011 Census: Key Statistics for England and Wales, March 2011. 2012. http://www.ons.gov.uk/ons/rel/census/2011-census/key-statistics-for-local-authorities-in-england-and-wales/stb-2011-census-key-statistics-for-england-and-wales.html Accessed: 28th November, 2016.

27 Nickel B, Barratt A, Hersch J, Moynihan R, Irwig L, McCaffery K. How different terminology for ductal carcinoma in situ (DCIS) impacts women's concern and management preferences: A qualitative study. Breast 2015;24:673–9.

28 Petrova D, Garcia-Retamero R, Cokely ET. Understanding the harms and benefits of cancer screening: a model of factors that shape informed decision making. Med Decis Making 2015;35:847-58.

29 Public Health England. Abdominal aortic aneurysm (AAA) screening, a free test for men aged 65 and over. 2015. https://www.gov.uk/government/publications/abdominal-

For peer review only - http://bmjopen.bmj.com/site/about/guidelines.xhtml

aortic-aneurysm-screening-invitation-leaflet Accessed: 28th November, 2016.

30 Public Health England. PSA testing and prostate cancer: advice for well men aged 50 and over. 2016. https://www.gov.uk/government/publications/prostate-specific-antigen-testing-description-in-brief Accessed: 28th November, 2016.

31 Smith SG, Raine R, Obichere A, Wolf MS, Wardle J, von Wagner C. The effect of a supplementary ('gist-based') information leaflet on colorectal cancer knowledge and screening intention: a randomized controlled trial. J Behav Med 2015;38:261–72.

32 Green MC. Narratives and cancer communication. J Commun 2006;56:S163–83.

33 Hinyard LJ, Kreuter MW. Using narrative communication as a tool for health behavior change: a conceptual, theoretical, and empirical overview. Health Educ Behav 2007;34:777–92.

For peer review only - http://bmjopen.bmj.com/site/about/guidelines.xhtml

Table 1 – Perceived clarity of overdiagnosis information: descriptive statistics for categorical/ordinal variables, adjusted ORs, 95% CIs, p-values for variables in the multivariable binary logistic regression model

		"How clear do you find this description of a risk of the test?" n (%)					Adjusted OR, 95% CI; p-value
Characteristic	Total (N=1,616)	Extremely clear (n=148; 9.2%)	Very clear (n=542; 33.5%)	Moderately clear (n=592; 36.6%)	Slightly clear (n=173; 10.7%)	Not at all clear (n=161; 10.0%)	Extremely/Very clear (vs. Not/Slightly/Moderately)
**Overdiagnosis information**							
Breast screening text	850	75 (8.8)	321 (37.8)	285 (33.5)	81 (9.5)	88 (10.4)	1.43, 1.17 to 1.75; **.001**
vs. Prostate screening text	766	73 (9.5)	221 (28.9)	307 (40.1)	92 (12.0)	73 (9.5)	
**Gender**							
Male	748	66 (8.8)	228 (30.5)	303 (40.5)	81 (10.8)	70 (9.4)	0.92, 0.74 to 1.14; .447
vs. Female	868	82 (9.4)	314 (36.2)	289 (33.3)	92 (10.6)	91 (10.5)	
**Ethnicity**							
White British	1,229	116 (9.4)	425 (34.6)	447 (36.4)	121 (9.8)	120 (9.8)	1.19, 0.92 to 1.54; .197
vs. Other ethnic groups	387	32 (8.3)	117 (30.2)	145 (37.5)	52 (13.4)	41 (10.6)	
**Marital status**							
Married or living as a couple	987	92 (9.3)	344 (34.9)	355 (36)	101 (10.2)	95 (9.6)	1.16, 0.93 to 1.45; .184
vs. Single, widowed, divorced,	629	56 (8.9)	198 (31.5)	237 (37.7)	72 (11.4)	66 (10.5)	

For peer review only - http://bmjopen.bmj.com/site/about/guidelines.xhtml

For peer review only

or separated							
**Highest level of education**							Overall p-value: .482
No formal qualifications	275	24 (8.7)	97 (35.3)	107 (38.9)	25 (9.1)	22 (8.0)	1.24, 0.86 to 1.79; .248
Approximately Level 1, 2, or 3	858	68 (7.9)	297 (34.6)	312 (36.4)	97 (11.3)	84 (9.8)	1.13, 0.87 to 1.46; .353
vs. Approximately Level 4	483						
**Social class grade**							Overall p-value: .610
Grade A or B	351	41 (11.7)	117 (33.3)	122 (34.8)	31 (8.8)	40 (11.4)	1.18, 0.85 to 1.64; .323
Grade C1 or C2	786	71 (9.0)	267 (34.0)	298 (37.9)	80 (10.2)	70 (8.9)	1.09, 0.85 to 1.40; .507
vs. Grade D or E	479	36 (7.5)	158 (33.0)	172 (35.9)	62 (12.9)	51 (10.6)	
**Personal diagnosis of cancer**							
Yes	86	11 (12.8)	36 (41.9)	25 (29.1)	6 (7.0)	8 (9.3)	1.34, 0.84 to 2.12; .218
vs. No	1,530	137 (9.0)	506 (33.1)	567 (37.1)	167 (10.9)	153 (10.0)	
**Knows someone with cancer**							
Yes	946	89 (9.4)	348 (36.8)	313 (33.1)	97 (10.3)	99 (10.5)	1.23, 0.99 to 1.53; .060
vs. No	670	59 (8.8)	194 (29.0)	279 (41.6)	76 (11.3)	62 (9.3)	
**Previously read a screening leaflet**							
Yes	907	101 (11.1)	340 (37.5)	298 (32.9)	87 (9.6)	81 (8.9)	1.35, 1.04 to 1.74; **.024**
vs. No	709	47 (6.6)	202 (28.5)	294 (41.5)	86 (12.1)	80 (11.3)	

For peer review only - http://bmjopen.bmj.com/site/about/guidelines.xhtml

**Previously read an NHS screening website**							
Yes	275	33 (12.0)	105 (38.2)	88 (32.0)	23 (8.4)	26 (9.5)	0.97, 0.72 to 1.30; .815
vs. No	1,341	115 (8.6)	437 (32.6)	504 (37.6)	150 (11.2)	135 (10.1)	
**Discussed screening with doctor/nurse**							
Yes	535	67 (12.5)	200 (37.4)	172 (32.1)	46 (8.6)	50 (9.3)	1.08, 0.83 to 1.39; .576
vs. No	1,081	81 (7.5)	342 (31.6)	420 (38.9)	127 (11.7)	111 (10.3)	
**Previously read or heard similar information**							
Yes	584	78 (13.4)	239 (40.9)	189 (32.4)	49 (8.4)	29 (5.0)	1.77, 1.40 to 2.23; **<.0005**
vs. No	1,032	70 (6.8)	303 (29.4)	403 (39.1)	124 (12.0)	132 (12.8)	

N.B. Adjusted ORs and 95% CIs are relative to a stated reference category; p-values <.05 are in bold; all predictor variables are included in the model

For peer review only - http://bmjopen.bmj.com/site/about/guidelines.xhtml

Table 2 – Perceived clarity of overdiagnosis information: descriptive statistics for continuous variables, adjusted ORs, 95% CIs, p-values for variables in the multivariable binary logistic regression model

		"How clear do you find this description of a risk of the test?" M (SD)					Adjusted OR, 95% CI; p-value
**Characteristic**	**Total (n=1,616)**	**Extremely clear (n=148; 9.2%)**	**Very clear (n=542; 33.5%)**	**Moderately clear (n=592; 36.6%)**	**Slightly clear (n=173; 10.7%)**	**Not at all clear (n=161; 10.0%)**	**Extremely/Very clear (vs. Not/Slightly/Moderately)**
**Age (in years)**	43.7 (15.7)	45.4 (14.8)	44.3 (16)	43.3 (15.9)	43.2 (15.7)	42.4 (14.7)	1.00, 0.99 to 1.00; .282
**Decision-making styles**							
Avoidant score	13.8 (3.9)	13.3 (4.4)	13.8 (3.8)	13.9 (3.9)	13.7 (3.9)	13.5 (3.8)	1.01, 0.98 to 1.04; .713
Dependent score	16.9 (3.5)	16.2 (4)	16.8 (3.5)	17.0 (3.4)	17.4 (3.5)	16.5 (3.4)	0.97, 0.94 to 1.00; .052
Intuitive score	18.7 (2.8)	18.8 (3.4)	18.9 (2.7)	18.6 (2.7)	18.7 (2.7)	18.3 (2.6)	1.02, 0.98 to 1.06; .413
Rational score	19.7 (2.6)	20.4 (2.7)	19.7 (2.6)	19.6 (2.6)	19.7 (2.8)	19.4 (2.6)	1.06, 1.02 to 1.11; **.009**
Spontaneous score	14.7 (3.7)	14.5 (4.1)	14.9 (3.6)	14.9 (3.6)	14.5 (3.8)	14.0 (3.7)	1.03, 1.00 to 1.07; .073

N.B. Adjusted ORs and 95% CIs are per unit increase; p-values <.05 are in bold; all predictor variables are included in the model

For peer review only - http://bmjopen.bmj.com/site/about/guidelines.xhtml

For peer review only

Figure Legends:

Figure 1 – Proportion of participants who perceived the information as extremely/very clear within each possible score level for rational and dependent decision-making styles (percentage and total number of participants in brackets)

For peer review only

For peer review only - http://bmjopen.bmj.com/site/about/guidelines.xhtml

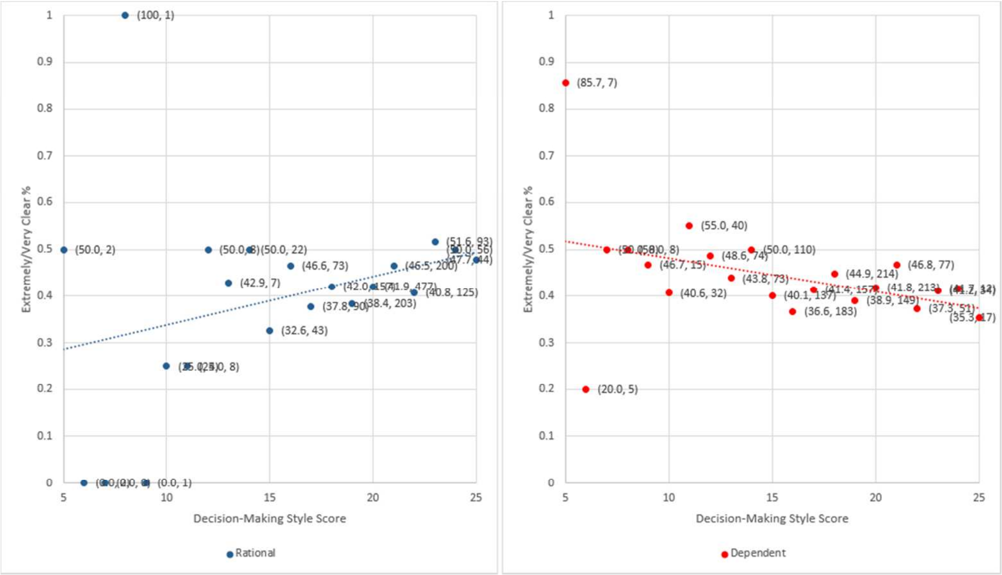

Figure 1 – Proportion of participants who perceived the information as extremely/very clear within each possible score level for rational and dependent decision-making styles (percentage and total number of participants in brackets)

84x48mm (300 x 300 DPI)

eview only

Appendix 1 – Sensitivity analyses: perceived clarity of overdiagnosis information: adjusted ORs, 95% CIs, p-values for categorical/ordinal variables in multivariable binary logistic regression models

	"How clear do you find this description of a risk of the test?" n (%)			
**Characteristic**	**Extremely (vs. less than extremely)**	**At least very (vs. less than very)**	**At least moderately (vs. less than moderately)**	**At least slightly (vs. not at all)**
	**Adjusted OR, 95% CI; p-value**	**Adjusted OR, 95% CI; p-value**	**Adjusted OR, 95% CI; p-value**	**Adjusted OR, 95% CI; p-value**
**Overdiagnosis information**				
Breast screening text vs. Prostate screening text	0.92, 0.65 to 1.30; .635	1.43, 1.17 to 1.75; **.001**	1.09, 0.85 to 1.40; .484	0.89, 0.64 to 1.24; .482
**Gender**				
Male vs. Female	1.03, 0.71 to 1.50; .873	0.92, 0.74 to 1.14; .447	1.20, 0.92 to 1.56; .183	1.24, 0.87 to 1.78; .243
**Ethnicity**				
White British vs. Other ethnic groups	1.26, 0.80 to 1.99; .312	1.19, 0.92 to 1.54; .197	1.35, 1.00 to 1.82; .054	1.17, 0.77 to 1.77; .464
**Marital status**				
Married or living as a couple vs. Single, widowed, divorced, or separated	0.98, 0.68 to 1.42; .909	1.16, 0.93 to 1.45; .184	1.12, 0.87 to 1.46; .382	1.03, 0.72 to 1.47; .879
**Highest level of education**	Overall: .535	Overall: .482	Overall: .075	Overall: .425

For peer review only

No formal qualifications	0.91, 0.50 to 1.66; .751	1.24, 0.86 to 1.79; .248	1.70, 1.07 to 2.68; .024	1.48, 0.80 to 2.75; .213
Approximately Level 1, 2, or 3	0.79, 0.52 to 1.20; .277	1.13, 0.87 to 1.46; .353	1.17, 0.86 to 1.59; .315	1.22, 0.82 to 1.84; .330
vs. Approximately Level 4				
**Social class grade**	Overall: .534	Overall: .610	Overall: .053	Overall: .260
Grade A or B	1.37, 0.79 to 2.38; .263	1.18, 0.85 to 1.64; .323	1.40, 0.95 to 2.07; .092	1.02, 0.61 to 1.70; .954
Grade C1 or C2	1.67, 0.75 to 1.83; .497	1.09, 0.85 to 1.40; .507	1.44, 1.07 to 1.94; .017	1.34, 0.89 to 2.02; .165
vs. Grade D or E				
**Personal diagnosis of cancer**				
Yes	1.23, 0.61 to 2.45; .565	1.34, 0.84 to 2.12; .218	1.20, 0.65 to 2.22; .557	1.03, 0.47 to 2.27; .934
vs. No				
**Knows someone with cancer**				
Yes	0.85, 0.59 to 1.23; .390	1.23, 0.99 to 1.53; .060	0.85, 0.65 to 1.11; .226	0.79, 0.55 to 1.13; .196
vs. No				
**Previously read a screening leaflet**				
Yes	1.30, 0.83 to 2.04; .257	1.35, 1.04 to 1.74; **.024**	1.12, 0.83 to 1.53; .461	1.11, 0.73 to 1.69; .619
vs. No				
**Previously read an NHS screening website**				
Yes	0.85, 0.54 to 1.36; .504	0.97, 0.72 to 1.30; .815	0.95, 0.65 to 1.39; .779	0.85, 0.51 to 1.42; .544
vs. No				

For peer review only

**Discussed screening with doctor/nurse**				
Yes	1.31, 0.87 to 1.97; .192	1.08, 0.83 to 1.39; .576	1.02, 0.74 to 1.41; .894	0.85, 0.55 to 1.30; .447
vs. No				
**Previously read or heard similar information**				
Yes	1.75, 1.19 to 2.57; **.004**	1.77, 1.40 to 2.23; **<.0005**	2.19, 1.61 to 2.98; **<.0005**	3.27, 2.08 to 5.14; **<.0005**
vs. No				

N.B. Adjusted ORs and 95% CIs are relative to a stated reference category; p-values <.05 are in bold; all predictor variables are included in each model with one exception (marked N/A in Appendix 2) due to a violation of the assumption linearity

For peer review only

Appendix 2 – Sensitivity analyses: perceived clarity of overdiagnosis information: adjusted ORs, 95% CIs, p-values for continuous variables in multivariable binary logistic regression models

Characteristic	"How clear do you find this description of a risk of the test?" n (%)			
	**Extremely (vs. less than extremely)**	**At least very (vs. less than very)**	**At least moderately (vs. less than moderately)**	**At least slightly (vs. not at all)**
	**Adjusted OR, 95% CI; p-value**	**Adjusted OR, 95% CI; p-value**	**Adjusted OR, 95% CI; p-value**	**Adjusted OR, 95% CI; p-value**
**Age (in years)**	1.00, 0.98 to 1.01; .642	1.00, 0.99 to 1.00; .282	1.00, 0.99 to 1.01; .329	1.00, 0.99 to 1.02; .562
**Decision-making styles**				
Avoidant score	1.01, 0.96 to 1.07; .711	1.01, 0.98 to 1.04; .713	1.03, 0.99 to 1.07; .143	0.99, 0.94 to 1.04; .611
Dependent score	0.94, 0.89 to 0.99; **.016**	0.97, 0.94 to 1.00; .052	0.98, 0.94 to 1.02; .289	1.04, 0.98 to 1.09; .176
Intuitive score	1.00, 0.94 to 1.07; .978	1.02, 0.98 to 1.06; .413	1.02, 0.98 to 1.07; .358	1.01, 0.94 to 1.07; .857
Rational score	1.15, 1.06 to 1.24; **.001**	1.06, 1.02 to 1.11; **.009**	1.03, 0.98 to 1.09; .215	1.08, 1.01 to 1.16; **.031**
Spontaneous score	1.02, 0.96 to 1.08; .574	1.03, 1.00 to 1.07; .073	N/A	1.09, 1.01 to 1.16; **.004**

N.B. Adjusted ORs and 95% CIs are per unit increase in the case of continuous variables; p-values <.05 are in bold; all predictor variables are included in each model with one exception (marked N/A) due to a violation of the assumption linearity

For peer review only

Appendix 3 - Numbers and percentages of participants excluded from the full sample of 2,111

**Excluded due to missing/inapplicable data on:**	**n (%)**
Reading a leaflet from the NHS about a screening test	39
Reading information about a screening test on an NHS website	15
Talking with a doctor or nurse about a screening test	18
Reading or hearing similar information about a screening test	74
Being diagnosed with cancer	20
Anyone close being diagnosed with cancer	13
Marital status	7
Education	216
Ethnicity	9
Perceived clarity of the description of a risk of the test	48
Decision-making style	36
**Total excluded**	**495 (23.4)**
**Total analysed**	**1,616**

For peer review only

STROBE Statement—checklist of items that should be included in reports of observational studies

	Item No	Recommendation	Checklist
**Title and abstract**	1	(*a*) Indicate the study's design with a commonly used term in the title or the abstract	P1-2
		(*b*) Provide in the abstract an informative and balanced summary of what was done and what was found	P2
**Introduction**			
Background/rationale	2	Explain the scientific background and rationale for the investigation being reported	P3-5
Objectives	3	State specific objectives, including any prespecified hypotheses	P4-5
**Methods**			
Study design	4	Present key elements of study design early in the paper	P5
Setting	5	Describe the setting, locations, and relevant dates, including periods of recruitment, exposure, follow-up, and data collection	P5-6
Participants	6	(*a*) *Cohort study*—Give the eligibility criteria, and the sources and methods of selection of participants. Describe methods of follow-up<br>*Case-control study*—Give the eligibility criteria, and the sources and methods of case ascertainment and control selection. Give the rationale for the choice of cases and controls<br>*Cross-sectional study*—Give the eligibility criteria, and the sources and methods of selection of participants	P5-6
		(*b*) *Cohort study*—For matched studies, give matching criteria and number of exposed and unexposed<br>*Case-control study*—For matched studies, give matching criteria and the number of controls per case	N/A
Variables	7	Clearly define all outcomes, exposures, predictors, potential confounders, and effect modifiers. Give diagnostic criteria, if applicable	P9-10
Data sources/ measurement	8*	For each variable of interest, give sources of data and details of methods of assessment (measurement). Describe comparability of assessment methods if there is more than one group	P6-10
Bias	9	Describe any efforts to address potential sources of bias	P9-10
Study size	10	Explain how the study size was arrived at	P6
Quantitative variables	11	Explain how quantitative variables were handled in the analyses. If applicable, describe which groupings were chosen and why	P9-10
Statistical methods	12	(*a*) Describe all statistical methods, including those used to control for confounding	P9-10
		(*b*) Describe any methods used to examine subgroups and interactions	P9-10
		(*c*) Explain how missing data were addressed	P9
		(*d*) *Cohort study*—If applicable, explain how loss to follow-up was addressed<br>*Case-control study*—If applicable, explain how matching of cases and controls was addressed<br>*Cross-sectional study*—If applicable, describe analytical methods	P9-10

For peer review only - http://bmjopen.bmj.com/site/about/guidelines.xhtml

		taking account of sampling strategy	
		(*e*) Describe any sensitivity analyses	P10
**Results**			
Participants	13*	(a) Report numbers of individuals at each stage of study—eg numbers potentially eligible, examined for eligibility, confirmed eligible, included in the study, completing follow-up, and analysed	P11
		(b) Give reasons for non-participation at each stage	Appendix 3
		(c) Consider use of a flow diagram	N/A
Descriptive data	14*	(a) Give characteristics of study participants (eg demographic, clinical, social) and information on exposures and potential confounders	P11; Table 1; Table 2
		(b) Indicate number of participants with missing data for each variable of interest	Appendix 3
		(c) *Cohort study*—Summarise follow-up time (eg, average and total amount)	N/A
Outcome data	15*	*Cohort study*—Report numbers of outcome events or summary measures over time	P11; Table 1; Table 2
		Case-control study—Report numbers in each exposure category, or summary measures of exposure	
		Cross-sectional study—Report numbers of outcome events or summary measures	
Main results	16	(*a*) Give unadjusted estimates and, if applicable, confounder-adjusted estimates and their precision (eg, 95% confidence interval). Make clear which confounders were adjusted for and why they were included	P11; Table 1; Table 2
		(*b*) Report category boundaries when continuous variables were categorized	Table 1; Table 2
		(*c*) If relevant, consider translating estimates of relative risk into absolute risk for a meaningful time period	N/A
Other analyses	17	Report other analyses done—eg analyses of subgroups and interactions, and sensitivity analyses	Appendix 1 Appendix 2
**Discussion**			
Key results	18	Summarise key results with reference to study objectives	P12-13
Limitations	19	Discuss limitations of the study, taking into account sources of potential bias or imprecision. Discuss both direction and magnitude of any potential bias	P14
Interpretation	20	Give a cautious overall interpretation of results considering objectives, limitations, multiplicity of analyses, results from similar studies, and other relevant evidence	P12-15
Generalisability	21	Discuss the generalisability (external validity) of the study results	P13-15
**Other information**			
Funding	22	Give the source of funding and the role of the funders for the present study and, if applicable, for the original study on which the present article is based	P15

*Give information separately for cases and controls in case-control studies and, if applicable, for exposed and unexposed groups in cohort and cross-sectional studies.

For peer review only - http://bmjopen.bmj.com/site/about/guidelines.xhtml

For peer review only

Note: An Explanation and Elaboration article discusses each checklist item and gives methodological background and published examples of transparent reporting. The STROBE checklist is best used in conjunction with this article (freely available on the Web sites of PLoS Medicine at http://www.plosmedicine.org/, Annals of Internal Medicine at http://www.annals.org/, and Epidemiology at http://www.epidem.com/). Information on the STROBE Initiative is available at www.strobe-statement.org.

For peer review only

For peer review only - http://bmjopen.bmj.com/site/about/guidelines.xhtml
